# Valorizing the Unexplored Filtration Waste of Brewing Industry for Green Silver Nanocomposite Synthesis

**DOI:** 10.3390/nano12030442

**Published:** 2022-01-28

**Authors:** Neha Venkatesh Rangam, Alcina Johnson Sudagar, Artur Ruszczak, Paweł Borowicz, József Tóth, László Kövér, Dorota Michałowska, Marek Łukasz Roszko, Krzysztof Robert Noworyta, Beata Lesiak

**Affiliations:** 1 Institute of Physical Chemistry, Polish Academy of Sciences, Kasprzaka 44/52, 01-224 Warsaw, Poland; aruszczak@ichf.edu.pl (A.R.); pborowicz@ichf.edu.pl (P.B.); knoworyta@ichf.edu.pl (K.R.N.); blesiak-orlowska@ichf.edu.pl (B.L.); 2 Institute for Nuclear Research, BemTér 18/c, H-4026 Debrecen, Hungary; toth.jozsef@atomki.hu (J.T.); kover.laszlo@atomki.hu (L.K.); 3 Institute of Agriculture and Food Biotechnology—State Research Institute, ul. Rakowiecka 36, 02-532 Warsaw, Poland; dorota.michalowska@ibprs.pl (D.M.); marek.roszko@ibprs.pl (M.Ł.R.)

**Keywords:** green chemistry, nanoparticles, waste valorization, antibacterial, silver chloride

## Abstract

The brewing industry generates a substantial amount of by-products rich in polyphenols, carbohydrates, sugars, sulfates, nitrogen compounds, organic carbon, and several elements, including chlorine, magnesium, and phosphorus. Although limited quantities of these by-products are used in fertilizers and composts, a large amount is discarded as waste. Therefore, it is crucial to identify different ways of valorizing the by-products. Research regarding the valorization of the brewery by-products is still in its nascent stage; therefore, it still has high potential. Herein, we report the valorization of the brewery by-product from the filtration stage of the brewing process (BW9) to synthesize silver nanocomposites as this waste has remained largely unexplored. The BW9 nanocomposites have been compared to those obtained from the brewery product B. The chemical composition analysis of BW9 and B revealed several organic moieties capable of reducing metal salts and capping the formed nanoparticles. Therefore, the brewery waste from stage 9 was valorized as a precursor and added to silver-based precursor at various temperatures (25, 50, and 80 °C) and for various time periods (10, 30, and 120 min) to synthesize silver nanocomposites. The nanocomposites obtained using BW9 were compared to those obtained using the main product of the brewing industry, beer (B). Synthesized nanocomposites composed of AgCl as a major phase and silver metal (Ag_met_) was incorporated in minor quantities. In addition, Ag_3_PO_4_ was also found in B nanocomposites in minor quantities (up to 34 wt.%). The surface morphology depicted globular nanoparticles with layered structures. Small ball-like aggregates on the layer representative of Ag_3_PO_4_ were observed in B nanocomposites. The surface of nanocomposites was capped with organic content and functional groups present in the brewery products. The nanocomposites demonstrated high antibacterial activity against *Escherichia coli* (*E. coli*), with BW9 nanocomposites exhibiting a higher activity than B nanocomposites.

## 1. Introduction

Beer is the third most consumed beverage globally, only behind tea and coffee [1]. The European brewing industry reached an eighty-year high of nearly 39.7 billion liters of beer with around 9500 breweries [2]. Poland was the third-largest beer producer, according to the 2017 statistics [3]. As the effluents from fermentation and filtering are high in organics/biochemical oxygen demand (BOD) and suspended solids, it is difficult to dispose of them in the environment [4]. Therefore, with growing concerns about environmental pollution, stricter legislations have enforced advanced treatment and recycling of the brewery industrial wastes. Waste treatment methods and alternate applications of the wastes have been explored in the past decade. Membrane filtration, quenched plasma, microbial fuel cells, nanofilters, combined aerobic and anaerobic treatment, and nanosorbents have been applied to treat brewery waste [4]. Converting the brewing waste into functional ingredients also has excellent potential, as explained by Corina Fărcaş et al. [5]. Application of brewery wastes in fish feed, agriculture, and as sorbents for heavy metals have been demonstrated [6,7]. However, there is still much scope in brewery wastes valorization.

**Scheme 1:**
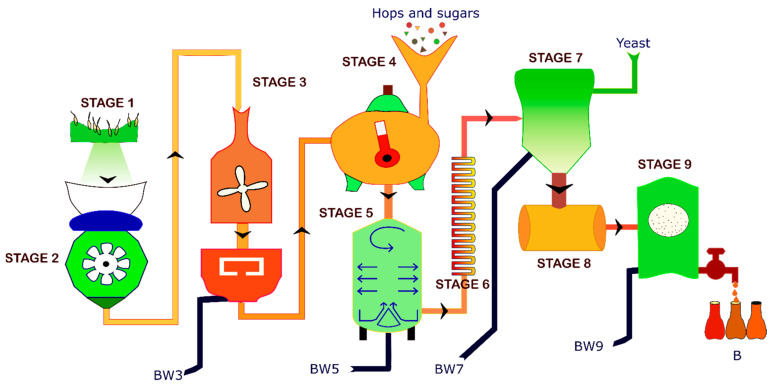
Brewing beer process and wastes produced in different stages.

Brewing is an extensive 10-step process (Scheme 1), resulting in waste production at different stages [8]. The process starts with malting, in which germination of fresh barley in water results in the formation of enzymes (amylase) responsible for the separation of starch. Following this, the malt is kilned between 71–82 °C and milled to various coarseness grades for better dissolution in water. Water is added to the milled malt in a mash-tun and mashed so that the starch dissolves and protein, sugar, and tannin are released. The sweet malt extract (wort) is separated from the solid substances called Brewer’s spent grains (BSG) in a lautering tun. The BSG is the first type of waste generated (BW3) in the brewing process, generally used as animal feed. The next stage involves boiling the wort, where the bitter and aromatic hops and sugar are added. During this step, the tannin and proteins are also separated. The wort is then clarified by feeding it into a whirlpool and tapping out the clear wort from the side. The undissolved hop particles and protein collected at the whirlpool center are the second type of waste (BW5) called wort precipitate. The clear wort is then cooled before fermentation. Alcoholic fermentation occurs when the added brewing yeast catalyzes the chemical conversion of wort sugars into alcohol and carbon dioxide. After the malt sugar is fermented, the yeast sinks and can be collected. This collected yeast forms the third type of waste (BW7) called Brewer’s spent yeast. After this, the beer is matured at low temperatures (1–2 °C), where the secondary fermentation process occurs, and the remaining yeast and protein-tannin sink to the bottom. Filtration separates the clarified beer from the fermentation waste and remaining hops using diatomaceous earth, forming the fourth type of waste (BW9).

The brewing industry product beer (B) and by-products BW3, BW5, and BW7 are rich in several compounds, including amino acids, carbohydrates, fatty acids, hop acids, polyphenols, proteins, and vitamins [5,9,10,11,12,13]. They are also enriched with sulfur-containing compounds responsible for imparting the peculiar taste in beers. In addition, calcium, chromium, copper, iron, magnesium, manganese, phosphorus, potassium, selenium, and zinc are detected in the by-products [5]. These by-products also have high nitrogen content. Although the by-product BW9 has not been studied in detail previously in the literature, it would also contain similar compounds as it is the waste from the brewing process’s final stage. As filtration is performed using diatomaceous earth, this component can also be expected in BW9. Medium-sized brewery generates monthly ~8 tons of dense sludge (diatomaceous earth with sediment) and approximately 5 to 7 times, i.e., 40–56 tons of wastewater, as BW9 waste. This sludge is utilized as soil fertilizers; however, the wastewater is discharged into the sewage lines. Although some brewery by-products’ composition and potential applications are discussed in the literature, filtration waste BW9 is often ignored. Therefore, as the filtration by-product from stage 9 (BW9) has not been explored yet, the prospective applications of this waste remain unknown. There is an immense potential for recycling the by-product BW9 from the brewing industry.

Waste-mediated synthesis of silver and silver-based nanoparticles has been researched in the past decades. Notably, household and industrial wastes have yielded biocompatible nanoparticles without chemical reducing agents [14]. Fruit peel, grape stalk waste, aquaculture, horticultural food waste, and sugar industry waste have been employed to obtain silver nanoparticles [15,16,17,18]. Biosynthesized nanoparticles have the advantage of eco-friendly synthesis, non-toxic effects, and a practically easy and cheap route. Silver and silver compound-based nanoparticles have been applied for cytotoxicity against cancer cells, antimicrobial and antibacterial activity, enhanced radiation effects, sensing, catalysis, and surface plasmon resonance effects, which have significantly extended the horizons in these fields [19,20,21,22]. Therefore, this work aimed to utilize the large amounts of waste produced in the brewery industry to convert silver salts to silver metal-based nanocomposites and study their chemical and physical characteristics. The research mainly focused on valorizing the fourth type of brewery waste, BW9, and its comparison to the final product, B, as a precursor in nanoparticle synthesis. Furthermore, the effect of the nanoparticle chemical composition on the bacterial growth of Gram-negative bacteria *Escherichia coli* has been explored.

Previously, we studied the nanocomposites prepared using brewery wastes from stages 5 (BW5) and 7 (BW7), wherein the silver nanocomposites contained high amounts of silver phosphate [23]. Disparately, the current study is focused on the silver nanocomposites containing majorly silver chloride synthesized using brewery waste from stage 9 (BW9) and the brewery product (B). Furthermore, comparing these nanocomposites to previously obtained ones yields significant information about the effect of the composition of precursors from the brewing industry on the nanocomposite formed.

## 2. Materials and Methods

### 2.1. Materials

Silver nitrate (AgNO_3_) was purchased from Avantor Performance Materials Poland S.A. company (former POCH S.A., Gliwice, Poland). Brewery waste was obtained from Jabłonowo Brewery, Wólka Kosowska, Poland S.A. The strong light beer of Jabłonowo Brewery was purchased from a commercial outlet. Brewery waste from stage 9 of the production line (BW9) or the final brewery product (B) was used as a precursor, and silver nitrate was the metal salt precursor.

### 2.2. Synthesis of Nanoparticles

Nanoparticles were synthesized using the previously reported method [23] via the one-pot synthesis methodology. The nanocomposites synthesized using the brewery by-product from the final stage of the production line, BW9, were compared to those obtained using brewery product B to observe the differences between the precursor solutions. First, the brewery waste BW9 was filtered using 150 mm filter paper. The brewery product B was used without any processing. The filtered brewery waste BW9 or the finished brewery product B was then stirred on a magnetic stirrer at 100 rpm to form a homogeneous mixture till they reached the required temperature (25, 50, and 80 °C). The salt precursor, i.e., silver nitrate, was added to the above solution to obtain a 100 mM concentration. After the required reaction times (10, 30, and 120 min), the solution was cooled for about 5–10 min to reach ~35 °C and then centrifuged at 10,000 rpm. After centrifugation, the supernatant was discarded, and the nanoparticles were washed several times with distilled water. Further, they were dried in the air oven at ~40 °C for 24 h. This dried powder was then ground, characterized, and used for further studies. The nanocomposites’ structure, elemental composition, surface morphology, and surface chemical composition were studied. Additionally, the antibacterial activity of nanocomposite was investigated to understand the effect of composition on bacterial growth.

### 2.3. Analysis of Brewery By-Product and Product

The brewery by-product BW9 and final product B were analyzed for the presence of elements, total carbohydrates, total polyphenols, total nitrogen, total fermenting sugars (fructose, glucose, maltose + sucrose, maltotriose), sulfate (SO_4_^2−^), total organic carbon (TOC), and other chemical compounds. The conventional combustion method was used to quantify most of the elements. The elemental content of chlorine was determined using the potentiometric-argentometric method. Total nitrogen content was determined using the Kjeldahl titration method (PN-A-04018:1975) [24]. Total polyphenol content was determined using the spectrophotometric method (PN-A-79093-13:2000) [25]. The total carbohydrates content was determined using the spectrophotometric method according to 9.26, 8.14 Analytica EBC, 2.7.3 MEBAK (2013) [26]. High-performance liquid chromatography equipped with refractometric detection (HPLC/RID) was used to determine the total fermenting carbohydrates content according to 9.27, 8.7 Analytica EBC, 2.7.2 MEBAK (2013) [27]. High-performance liquid chromatography equipped with conductometric detection (HPLC/CD) was used to determine sulfate content according to 2.22.1 MEBAK (2013) [28]. The spectrometric method determines the total organic carbon according to CLA/SR/26/2012 [29].

### 2.4. Analysis of Nanocomposites

Fourier transform infrared spectra (FT-IR) were recorded using the Fourier Vertex 80 V spectrophotometer equipped with Platinum ATR (Bruker Inc., Billerica, MA, USA) in Attenuated Total Reflectance (ATR) mode at the pressure below 5 hPa. FT-IR yields information about the chemical groups present in the sample. Liquid samples were dried prior to the measurements. The apparatus parameters such as the full width at half-maximum (FWHM) of 2 cm^−1^ and number of scans 1024 were used to record the spectra. X-ray diffractograms were recorded using an Empyrean X-ray diffractometer (Bruker Inc., Billerica, MA, USA) for testing polycrystalline materials with equipment enabling measurements in Bragg–Brentano geometry. It contained an X-ray tube with Kβ filters, the goniometer in the *θ*-*θ* vertical system and the Cu Kα radiation source. X’pertHighScore Plus software (Malvern Panalytical, Malvern, UK) matches the peaks to reference patterns in the International Centre for Diffraction Data (ICDD) and determines the chemical compound and its phase in the sample. Elemental analysis was performed using PANalytical MiniPal 4 PW4025/00 energy dispersive X-ray fluorescence spectrometer (EDXRF) (PANanalytical Inc., Malvern, UK) that provides quantitative information about the elements present in the sample. Morphology was determined using images recorded by the ’FEI Nova NanoSEM 450’ (FEI, Hillsboro, OR, USA) scanning electron microscope (SEM). The images were recorded at 10 kV high voltage in immersion mode. Secondary electron detection was performed for image processing. An energy-dispersive X-ray analyzer (EDX) installed along with SEM was used for elemental mapping of nanocomposites surface.

The X-ray photoelectron spectroscopy (XPS) spectra were measured using the ESA-31 electron spectrometer in ultra-high-vacuum (custom built) equipped with a home-made X-ray excitation source (Al X-rays Kα λ = 8.3 Å), a hemispherical electron energy analyzer with a 0.5% energy resolution without retardation, an electron gun (LEG62-VG Microtech, Vacuum Generators, East Grinstead, UK), and argon (Ar^+^) ion source of AG21 (VG Scientific, Vacuum Generators, East Grinstead, UK) [30]. The XPS measurements were performed using fixed retarding ratio (FRR) mode (k = 4, 8, 16) at a photon incidence and electron emission angles of 70° and 0°, respectively, with respect to the surface normal of the specimen. XPS/AES QUASES simple backgrounds, ver. 2.2, 1999–2001 (Tougaard Inc., Odense SO, Denmark) was used for background subtraction [31]. After Tougaard inelastic background subtraction, all the photoelectron spectra were fitted to Gaussian-Lorentzian asymmetric components using the XPS peak fitting program for WIN95/98 XPSPEAK, ver. 4.1 (XPSPEAK4.1, R W M Kwok, Shatin, Hong Kong) [32]. Multimodel XPS quantification program for 32-bit Windows, XPS MultiQuant, ver. 7, 1999–2001 (M. Mohai, Budapest, Hungary) was used for quantification [33,34].

### 2.5. Bacterial Susceptibility Tests

The Gram-negative strain *Escherichia coli* ATCC 25,922 was used to test the antibacterial activity of nanocomposites synthesized at 25 and 80 °C. The study was performed as reported previously [23]. The bacterial strain was grown for 24 h and then added to dilutions of ultrasonicated nanocomposites to get the final bacterial concentration of 5 × 10^5^ CFU mL^−1^. Different nanocomposite concentrations (0.8, 1.6, 3.125, 6.25, 12.5, 25, and 50 μg mL^−1^) were incubated with inoculum for 24 h. The cell viability assay using alamarBlue reagent was performed following 24 h incubation for determining minimum inhibitory concentration (MIC). Subsequently, nanocomposite concentrations higher than the MIC were incubated for 24 h with bacterial inoculum, and 10 μL was pipetted on the agar plates to determine the minimum bactericidal concentration (MBC). The concentration at which no bacterial colonies were present on the agar plates after 24 h incubation was considered MBC. Finally, sub-MIC, MIC and MBC concentrations of nanocomposites were added to bacterial inoculum to study time-kill kinetics. The 10 μL volume of the dilutions obtained between 0 and 24 h were inoculated on agar plates. The final *E. coli* concentration in the solution was calculated using the number of colonies grown on agar plates. Bacterial culture without nanocomposites was used as a positive control.

## 3. Results

### 3.1. Characterization of Brewery By-Product and Product

The brewery by-product BW9 and product B were characterized in detail to understand their composition (Table 1). BW9 is the last stage of waste produced in the brewery production line before packaging the final product B; thus, it contains only tiny amounts of filtrate material diluted in large quantities of water. Product B, therefore, is noticeably richer in polyphenols, carbohydrates, sugars, nitrogen, and sulfates than BW9. Subsequently, the total organic carbon (TOC) content in B is also significantly higher than in BW9. The brewery product B has a large quantity of glucose sugar in the composition. In contrast, BW9, differently, contains low quantities of sugar. Similar differences have been obtained for carbohydrates where BW9 is almost deficient in carbohydrates and B has significant quantities. The brewery precursors are rich in potassium, phosphorous, carbon, magnesium, and sodium. Iron is also found in the agents. BW9 is richer than B only in calcium and iron content. Traces of manganese and aluminum are also observed in the BW9 and B.

**Figure 1:**
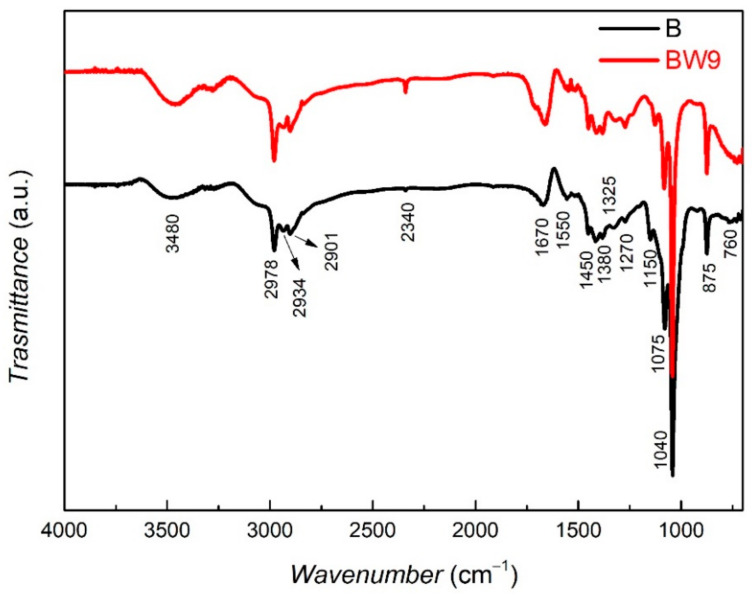
FT-IR spectra of brewery by-product BW9 and product B.

The FT-IR spectrum of the brewery waste BW9 is almost similar to that of the main product B (Figure 1). Both precursors contain a variety of organics. The broad band around 3600–3300 cm^−1^ can be associated with the intermolecular H-bonded O–H stretching in polyphenols as a strong vibration combined with a secondary amine NH stretching (a weak vibration) or hydrogen-bonded OH and NH groups. Further, several bands associated with alkane C–H stretches can also be observed in the range between 2990–2850 cm^−1^. Amide I, in particular: C=O stretching and amide II, in particular: R–NHR’, NH_2_-scissoring [35] and N–H bending, as well as C=N stretching can be observed at 1670 cm^−1^ and 1550 cm^−1^, respectively [36,37]. C–H alkane bending vibrations were observed around 1380 and 1450 cm^−1^. Sharp peaks at around 1075 and 1040 cm^−1^ can be associated with the C–O vibrations. The peaks at 1040 and 1270 cm^−1^ could represent the C–O vibration of the C–OH group and the C–C stretch in the carbohydrate structure [38] or the C–N stretching observed in the range 1250−1020 cm^−1^. The peak at 1110 cm^−1^ could correspond to the C–O band stretching of the C–O–C linkage. The 1150 cm^−1^ vibration can also represent the C–O bending of the C–OH groups in carbohydrates [39]. X–H bending out-of-plane (X = C, O, N) or NH_2_ rocking and wagging can be observed below 1000 cm^−1^. In particular, the sharp peak at around 875 cm^−1^ can be affiliated with −OH bending vibrations of alcohol, based on the observation from a previous study wherein it was found that the brewery waste obtained after the fermentation stage (BW7) only exhibited this peak [23]. Therefore, this peak can also be observed in BW9 and B as they are obtained after this fermentation stage 7. The range of vibrations obtained in the IR region indicates that the brewery by-product and product are rich in polyphenols, carbohydrates, lipids, and proteins [13,36,37,38,39,40,41,42]. Therefore, this rich composition of the brewery by-product and product should efficiently convert the metal salts to yield capped green nanoparticles.

### 3.2. Characterization of Nanocomposites

#### 3.2.1. Crystallography and Phase Analysis

**Figure 2:**
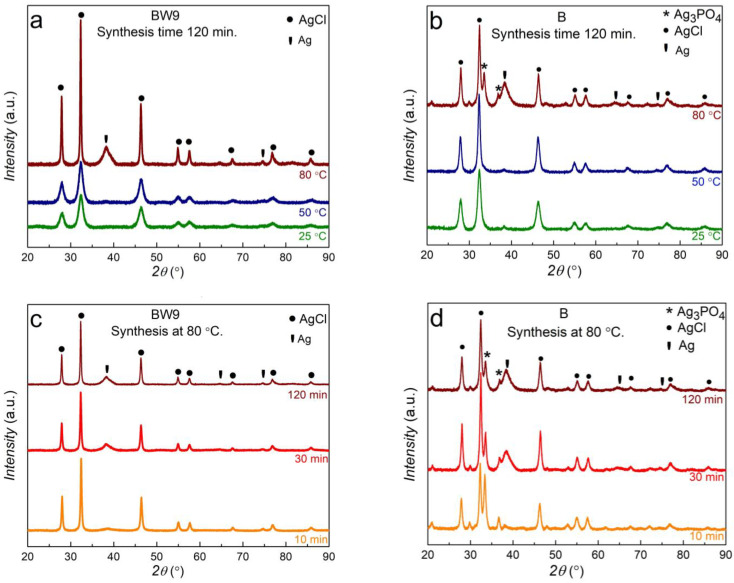
X-ray diffractograms of nanocomposites synthesized at different temperatures and times using (**a**,**c**) brewery by-product BW9 and (**b**,**d**) brewery product B.

The XRD diffractograms of BW9 and B nanocomposites synthesized under conditions of different temperatures showed reflexes characteristic for silver chloride AgCl (ICDD 98-005-6538) and metal silver Ag_met_ (ICDD 98-060-4629) phases (Figure 2). Traces of silver phosphate Ag_3_PO_4_ (ICDD 98-002-7843) were also found in samples prepared using B. The phase composition was evaluated from the most intensive XRD reflexes representing every phase, i.e., (111) at 2*θ* = 38.1° for Ag, (012) at 2*θ* = 33.3° for Ag_3_PO_4_, and (002) at 2*θ* = 32.2° for AgCl. The phase content was evaluated using the Reference Intensity Ratio (RIR) values with respect to corundum [43]. Scherrer’s equation determined the average nanocrystallite size for each phase and standard deviation for phases with more than one reflex over the range of 2*θ* from 20 to 60°. The results are listed in Table 2. Overall, with increasing synthesis temperature and time, the crystallinity of the nanomaterials increases. The peaks become narrower, indicating the formation of larger particles promoted by higher synthesis temperature and time.

BW9 waste-mediated synthesis led to the formation of pure AgCl nanoparticles (73.2–100 wt.%), and at synthesis temperatures above 50 °C, Ag_met_ (3–26.8 wt.%) was also incorporated in the structure (Figure 2a,c). The characteristic AgCl reflexes are observed at 27.8° (111), 32.3° (002), 46.3° (022), 54.9° (113), 57.5° (222), 67.5° (004), 74.5° (133), 76.8° (024), and 85.8° (224). The silver chloride has crystallized into a face-centered cubic NaCl-like structure with space group Fm-3m. Ag_met_ phase content increases at the cost of the AgCl phase with increasing synthesis temperature. As the synthesis temperature is increased, growth of Ag_met_ nanoparticle phases is evidenced through the peaks occurring at 2*θ* values 38.1° (111), 64.5° (022), and 77.4° (113). These peaks attributed to cubic structured silver nanoparticles strongly depend on the synthesis temperature. The Ag_met_ phase increases with an increase of synthesis time and temperature at the cost of the AgCl phase. The crystallite size (Table 2) of the silver chloride nanoparticles increases from 5.6 ± 0.5 nm for room temperature (25 °C) synthesis to 19.4 ± 3.1 nm for high temperature (80 °C) synthesis while that of Ag nanoparticles remained almost constant. This indicates that increasing synthesis temperature favors the growth of AgCl nanoparticles over nucleation.

The finished beer product (B), disparately, resulted in a mixed composite of AgCl, silver orthophosphate (Ag_3_PO_4_), and Ag_met_ nanoparticles (Figure 2b,d). The primary phase is still AgCl which decreases from 94 wt.% at room temperature synthesis to 51.8 wt.% for synthesis at 80 °C. The identified Ag_met_ and AgCl nanoparticles exhibit a cubic structure similar to those obtained from BW9. With increasing synthesis temperature from 25 °C to 50 °C, the phase content of AgCl increases. At 80 °C, Ag_3_PO_4_ and Ag metal phases increase at the cost of the AgCl phase in the nanocomposite. This suggests that the PO_4_^3−^ could be a part of the organic composition of precursors released at elevated temperatures, which combines with Ag^+^ ions to form Ag_3_PO_4_. The crystallite size trend for AgCl in B nanocomposites is similar to that observed for BW9 nanocomposites, i.e., it increases from 6.1 ± 1 to 10.7 ± 1.2 nm with increasing synthesis temperature. A strong dependence of Ag_met_ nanoparticle growth on synthesis temperature is observed when using B. The crystallite size of Ag_met_ nanoparticles decreases from 10.7 nm at 25 °C to 3 nm at 80 °C, unlike Ag nanoparticles with constant crystallite size in BW9 nanocomposites. Reflections at 20.9° (011), 33.3° (012), and 36.6° (112) have been assigned to Ag_3_PO_4_. The Ag_3_PO_4_ nanoparticles are face-centered cubic consisting of isolated tetrahedrons of AgO and PO_4_ connected by corner oxygen atoms. Although very few reflections were characteristic for Ag_3_PO_4_, previous research on nanocomposites obtained using brewery wastes from stages 5 and 7 confirms this phase’s occurrence in brewing industry product-mediated synthesis [23]. The size and phase content of these Ag_3_PO_4_ nanoparticles are the highest at the synthesis temperature of 80 °C. Low silver oxides content could not be observed in XRD analysis. It can be concluded that increasing synthesis temperature favors growth over nucleation of AgCl and Ag_3_PO_4_ nanoparticles, while nucleation is favored for Ag_met_ nanoparticles.

The peaks attributed to cubic structured silver nanoparticles also show a strong dependence on the synthesis time. At synthesis temperature of 80 °C, the synthesis time plays a significant role in the growth of metallic silver nanoparticles as it evolves with time at the cost of the AgCl phase for BW9 and B nanocomposites (Table 2). BW9 nanocomposites show increasing incorporation of Ag nanoparticles with time. At this synthesis temperature, the Ag_3_PO_4_ phase content in B nanocomposites is highest after 10 min of synthesis time, the AgCl phase content decreases, and that of Ag_met_ increases with synthesis time. The crystallite size of the AgCl and Ag nanoparticles increases with the synthesis time for BW9 nanocomposites, indicating that synthesis using BW9 favors the growth of nanoparticles over nucleation with increasing synthesis time. By contrast, in B nanocomposites, the crystallite size for Ag_3_PO_4_ and AgCl nanoparticles also increases, but the Ag_met_ nanoparticles’ crystallite size decreases with increasing synthesis time. This implies that at 80 °C, the increasing synthesis time favors growth over nucleation for Ag_3_PO_4_ and AgCl nanoparticles; varyingly, the increasing synthesis time favors nucleation over the growth of Ag_met_ nanoparticles. Thus, increasing synthesis time favors the incorporation of Ag_met_ into the nanocomposites, and depending on the precursor used, the growth or nucleation favors. For AgCl and Ag_3_PO_4_ nanoparticles, the increasing time favors growth over nucleation.

#### 3.2.2. Elemental and Morphological Analysis

The EDXRF (Energy Dispersive X-ray Fluorescence) method used to analyze the elemental composition of the samples indicated the composition of silver (Ag), chlorine (Cl), phosphorus (P), and sulfur (S) in all the nanocomposites. The weight percentage of these elements was calculated and presented in Table 3. Weight percentage (wt.%) of Ag increases, and that of Cl decreases with synthesis time and increasing synthesis temperature for BW9 nanocomposites. This decrease of Cl is consistent with the observed increase in the Ag phase obtained by the XRD of BW9 nanocomposites (Table 2). P and S are present only in trace amounts in BW9 nanocomposites, indicating they could be a part of the carbonaceous shell. For B nanocomposites, the weight percentage of Ag decreases from 25 to 50 °C and then increases at 80 °C. This trend of Ag can be attributed to the growth of the Ag_3_PO_4_ phase observed in the XRD analysis for B nanocomposites. At the same time, the Cl shows inverse behavior for the increasing synthesis temperature (increases from 25 to 50 °C and then decreases at 80 °C) and time (increases from 10 to 30 min and then decreases at 120 min. P (wt.%) increases with synthesis temperature in B nanocomposites concurrent with the increase in the Ag_3_PO_4_ phase observed in XRD. However, at 80 °C synthesis temperature, the P content is highest after 10 min synthesis time and decreases for higher synthesis time. This observation also complies with the Ag_3_PO_4_ phase growth in XRD. S present in trace amounts could be a part of the organic coating over the nanoparticles. However, Ag_3_PO_4_ phase content (Table 2) is not directly comparable to the P content (Table 3) in BW9 and B nanocomposites, implicating the presence of different chemical forms of P, possibly in the organic overlayer.

**Figure 3:**
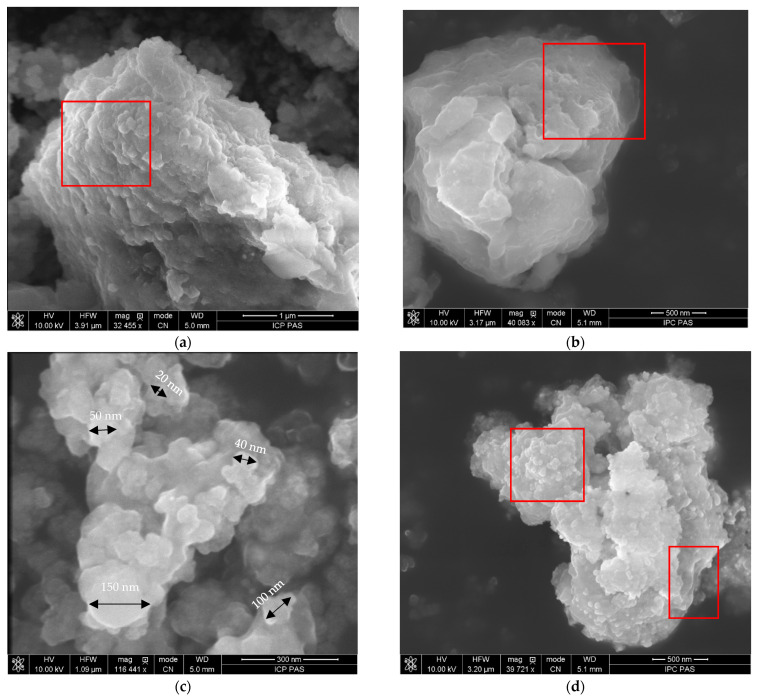

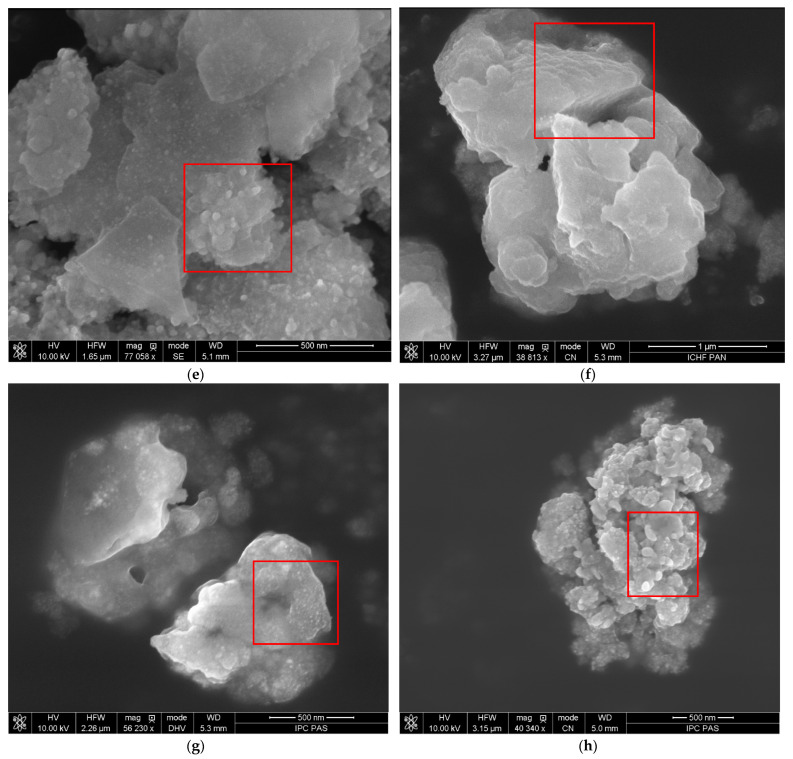

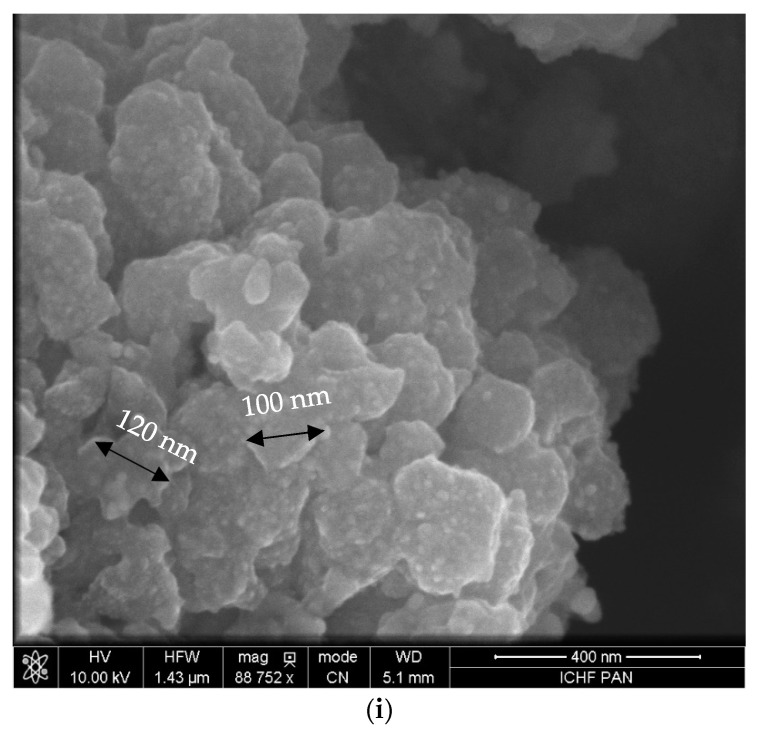
SEM images of nanocomposites synthesized after 120 min using brewery waste BW9 at (**a**–**c**) 25 °C and (**d**,**e**) 80 °C, and product B at (**f**,**g**) 25 °C and (**h**,**i**) 80 °C.

AgCl nanoparticles in the BW9 nanocomposites synthesized at 25 °C (BW9Ag1) showed an aggregated flakey layered morphology marked in the red square (Figure 3a–c). Aggregates of nanoparticles in the organic shell have a diameter ranging from 20 to 150 nm (Figure 3c, black arrows). With the increasing synthesis temperature to 80 °C (BW9Ag3), as Ag_met_ nanoparticles got introduced, the morphology slightly changed to a mixture of layers and globular structures marked in red (Figure 3d,e). B nanocomposites synthesized at 25 °C (BAg1) exhibited a combination of layered structures of AgCl, and smaller balls representative of Ag_3_PO_4_ marked in the red square fused to form a uniform composite structure (Figure 3f,g). These balls were similar to previously reported structures in BW7 nanocomposites containing majorly Ag_3_PO_4_ [23]. As the synthesis temperature was increased to 80 °C (BAg3), the growth of Ag_3_PO_4_ and Ag nanoparticles changed the morphology to aggregated platelet structures made of tiny balls and globules marked in the red square (Figure 3h,i). The elemental distribution of Ag, Cl, and P on the nanocomposites’ surface obtained using elemental mapping (Figure S1a–d in Supplementary Materials) indicates that the elements are uniformly distributed over the surface. This implies that the nanoparticles’ morphological changes in shapes and sizes are due to incorporating certain phases in the structure; however, these phases are not present independently but rather as a mixed phase composite.

#### 3.2.3. Chemical Analysis

**Figure 4:**
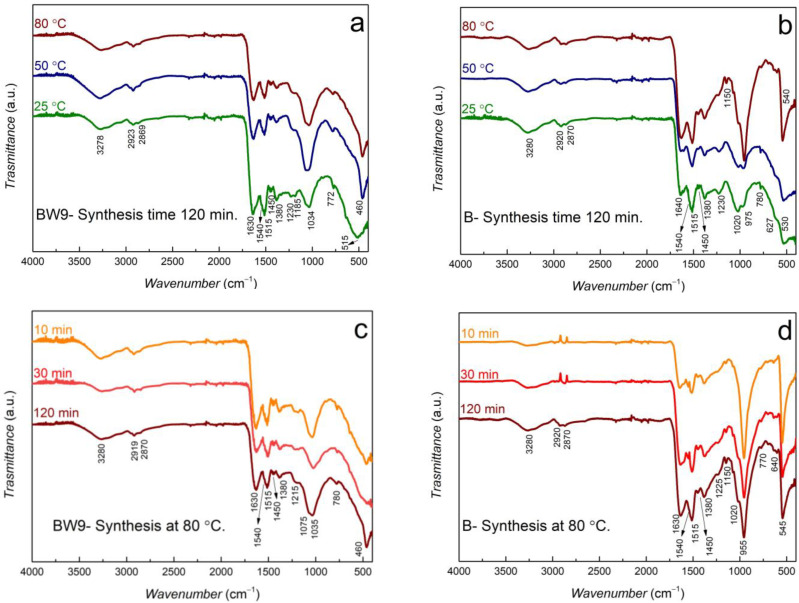
FT-IR spectra of (**a**,**c**) BW9 and (**b**,**d**) B nanocomposites at different temperatures and times.

The broad peak observed between ~3500–3000 cm^−1^ frequency indicates the stretching vibrations of the hydrogen-bonded OH group in nanocomposites synthesized using BW9 and B (Figure 4). The broad band can also be attributed to the P–OH bond in B nanocomposites where Ag_3_PO_4_ is present in minor quantities. Furthermore, the N–H stretching vibrations in amide can also be assigned to this broad band. This band has shifted considerably to lower frequencies in nanocomposites than the precursors BW9 and B (Figure 1), indicating a complexation of the functional groups with the nanoparticles. The peaks observed between 2860–3000 cm^−1^ belong to symmetric and asymmetric C–H stretching vibrations from alkanes in various chemical environments. These vibrations are weak in the nanocomposites, although significant in the waste BW9 and product B (Figure 1), similar to that observed in our previous BW7 nanocomposites [23]. The stretching vibrations of amide I and II around frequencies centered between 1630–1640 cm^−1^ could be attributed to strong C=O stretching and medium N−H bending. The vibration at 1515 cm^−1^ assigned to N−H bending has been observed in all nanocomposites [44]. The 1380 and 1450 cm^−1^ bending vibrations of CH_2_ and CH_3_ deformations in alkanes are also evident in nanocomposites. These bands are broader for nanocomposites than for precursors indicating a variety of chemical environments. C–O vibrations are also visible around 1035 cm^−1^ for BW9 nanocomposites. In B nanocomposites, this vibration is coupled to the PO_4_^3−^ vibration and, therefore, shifted to lower frequencies of about 1020 cm^−1^. These vibrations of the functional groups indicate that the organics from BW9 and B precursors have successfully capped the nanoparticles. However, the capping is different depending on the precursor used. The shifts in the vibration frequencies of the functional groups in nanocomposites (Figure 4) compared to that observed in precursors BW9 and B (Figure 1) confirm the complexation of nanoparticles to the functional groups.

The broad peak observed at frequencies of around 460–550 cm^−1^ represents the Ag–O stretch modes. However, this mode is also characteristic of the bending vibration of O=P–O in B nanocomposites [45,46]. Therefore, the presence of the Ag_3_PO_4_ phase intensifies the peak centered around 550 cm^−1^ in these B nanocomposites (Figure 4b,d). The vibration around this region is broad in BW9 nanocomposites, indicating that these materials do not have Ag_3_PO_4_ in the structure. There is a possibility of the presence of silver oxides in the nanocomposites because a broad band is still present. The vibration mode observed around 955–970 cm^−1^ is characteristic of P–O asymmetric stretching vibrations of the phosphate (PO_4_^3–^) [45,46,47]. These modes of PO_4_^3−^ are intensified in B nanocomposites at high synthesis temperatures, providing evidence of Ag_3_PO_4_ growth. These vibrations are absent in BW9 nanocomposites. The characteristic vibration modes for Ag_3_PO_4_ in B nanocomposites are relatively more intense for 80 °C syntheses after 10 min, concurrent with the highest Ag_3_PO_4_ content obtained in XRD analysis (Table 2).

The surface content of elements in the investigated samples was evaluated from the peak areas of Ag 3d_5/2–3/2_, C 1s, N 1s, O 1s, P 2p, S 2p, B 1s, and Cl 2p photoelectron lines (Figure S2 in Supplementary Materials) after Tougaard background subtraction [31]. The XPS MultiQuant software [34] accounting for Scofield photoionization cross-sections [48], inelastic electron scattering (IMFP: inelastic mean free path), and analyzer transmission function was applied as well. The quantification of surface elemental composition is presented in Table 4. In BW9 nanocomposites, the resulting surface elementary content expressed in wt.% shows predominantly Ag, C, O, and a smaller amount of Cl, N, S, and B (only at 25–50 °C in 120 min synthesis). In B nanocomposites, additionally, P is present. The ratio of C to Ag in BW9 nanocomposite is larger than the respective B nanocomposites (Figure S3a in Supplementary Materials), indicating a thicker organic overlayer consisting of carbon and C–O groups. In BW9 nanocomposites, the ratio of C to Ag increases with synthesis temperature and is more stable for time dependence at 80 °C, whereas in B nanocomposites, the ratio is stable with temperature and increases with time at 80 °C. Similarly, the decreasing ratio of C to O with synthesis temperature and time is observed (Figure S3b in Supplementary Materials), indicating overlayer enrichment with oxygen, where oxygen may be engaged into Ag oxide forms and surrounding organic material. The ratio of Ag to O decreases with temperature and time of synthesis at 80 °C (Figure S3c in Supplementary Materials). The surface (XPS analysis) and bulk (EDXRF analysis) elementary content of Ag, P, Cl, and S normalized to 100% with increasing Ag and decreasing P, Cl, and S content under temperature and time of synthesis (Figure S4 in Supplementary Materials) indicates no remarkable differences.

For evaluating the chemical state of elements present, the spectra were binding energy (BE) calibrated. The calibration of the C 1s, O 1s, Ag 3d_5/2-3/2_, N 1s, P 2p, Cl 2p, S 2p, and B 1s spectra was performed according to Ag 3d_5/2-3/2_ spectra predominant state revealed in the XRD patterns (Table 2). The following BE values for different Ag chemical states were applied: 367.8 eV for Ag_3_PO_4_ [49,50], 368.1 eV for AgCl [51], and 368.3 eV for Ag_met_ [52,53]. The oxidized Ag structures like Ag oxides (AgO, Ag_2_O) and AgOH were reported as shifted towards smaller BE values from Ag_met_ by 0.5–0.6 eV (Ag_2_O) and by 0.9–1 eV (AgO and AgOH). Similarly, the BE shift towards smaller BE values from Ag_met_ has been previously observed for Ag–glucose structures [54]. However, to optimize the fitting conditions, an additional peak was considered at BE smaller than that characteristic for Ag oxidized forms. The fitting conditions proceeded for constant ratios of full width at half maximum (FWHM) of peaks representative for each chemical form and no constrain for BE value of additionally assumed peak. The results of the applied fitting procedure representing the content of different chemical forms are presented in Table 5 and Figure S5a in Supplementary Materials.

The surface of both BW9 and B nanocomposites indicates a predominantly existing AgCl chemical form. The chemical state observed at 365.4 ± 0.3 eV (BW9 nanocomposites), and 364.9 ± 0.4 eV (B nanocomposites) can be interpreted as Ag oxidized forms linked with organic material through hydrogen bonding that results in the formation of a kind of organometallic structure denoted as AgO-polymer form. The content of the AgO-polymer to Ag oxidized forms is smaller in BW9 nanocomposites due to probably smaller amounts of polyphenols, carbohydrates, and fermentable sugars present in BW9 brewery waste. Comparison of content of silver phase at the surface (XPS) and in bulk (XRD) indicates similar behavior under temperature and time of synthesis for BW9 and B nanocomposites. However, no Ag_met_ and Ag_3_PO_4_ at the surface were observed, except for B nanocomposite synthesized at 80 °C after 10 min (BAg5) (Figure S6 in Supplementary Materials). With increasing synthesis temperature and time, the AgCl phase content on the surface decreases slightly, whereas the total AgCl phase in bulk decreases significantly (Table 2). The decrease of AgCl on the surface is accompanied by increasing content of Ag oxides and AgO-polymer phase.

The fitting results of C 1s spectra (Table 6, Figure S5b in Supplementary Materials) indicate the following BE values for the obtained carbon chemical forms: 285.3 ± 0.1 eV for the C sp^3^ hybridizations, 284.4 ± 0.1 eV for the C sp^2^ hybridizations, 286.2 ± 0.1 eV for hydroxyl group (–C–OH), and 287.3 ± 0.1 eV for carbonyl group (–C=O), what remains in agreement with a literature data [55]. No carboxyl group (–COOH) is present at the surface. The following BE values were obtained for the O 1s line: 530.77 ± 0.14 eV for Ag–O chemical form, 531.81 ± 0.4 eV for C=O and 532.41 ± 0.44 eV for C–O [55,56]. The content of oxygen groups (C–OH, C=O) and Ag–O chemical state resulting from the fitting of O 1s spectra agrees with the respective content resulting from the fitting of Ag 3d_5/3-3/2_ (Ag–O) and C 1s (C–OH, C=O) spectra. The resulting fitted O 1s spectra are shown in Figure S5c Supplementary Materials. The content of carbon-oxygen groups varies with nanomaterial and synthesis conditions (Table 6). The nanocomposites synthesized using B, which is rich in polyphenols, carbohydrates, and fermentable sugars, show higher content of oxygen groups, especially C=O groups. The content of oxygen-rich groups increases with temperature and time of synthesis. In BW9 nanocomposites, the increasing synthesis temperature and time pride larger content of C–OH groups on account of C=O groups. The overlayer thickness on Ag nanocrystallites listed in Table 6 is evaluated from the Ag 3d_5/2-3/2_ spectra, including an inelastic background in the vicinity of this photoelectron transition using the QUASES-Analyze [57] Buried Layer (BL) model and the inelastic mean free path value of Ag 3d_5/2-3/2_ photoelectrons in carbon, i.e., 3.1 nm, according to the TPP-2M formula [58]. The results of spectra evaluation are present in Figure S7a,b in Supplementary Materials.

The BE values of N 1s, Cl 2p, P 2p, S 2p, and B 1s photoelectron lines were evaluated after binding energy scale calibration with respect to Ag 3d_5/2-3/2_ photoelectron transition and averaged for BW9 and B nanocomposites prepared at different synthesis temperatures and times. These BE values confirm the presence of elements in the form of chemical compounds. Nitrogen (N 1s BE = 399.5 ± 0.1 eV) indicates amino group (C–NH_2_) present in a protein structure [59]. Chlorine (Cl 2p BE = 198.5 ± 0.1 eV) is identified as AgCl [59]. The phosphorous (P 2p BE = 133.3 ± 0.1 eV) present in B nanocomposites shows the oxidized form of phosphate [58]. The boron present in BW9 nanocomposites synthesized between 25–50 °C and B nanocomposites show the averaged value of BE = 188.5 ± 0.15 eV, which may be ascribed to boron in the organic matrix, i.e., B_x_(CH)_y_ [59]. Sulfur shows two forms, i.e., at BE = 163.1 ± 0.05 eV indicating sulfur in the organic environment, and BE = 168.3 ± 0.15 eV indicating sulfur in the oxygen-rich organic environment [59]. Both forms of sulfur occur in BW9 and B nanocomposites for different synthesis temperatures and times. BW9 nanocomposites show a higher content of sulfur at 168.25 ± 0.11 eV than B nanocomposites.

#### 3.2.4. Bacterial Susceptibility Testing

The bacterial susceptibility test was performed against *E. coli* for nanocomposites synthesized at 25 °C and 80 °C for 120 min (BW9Ag1, BW9Ag3, BAg1, and BAg3). The nanocomposites synthesized at different temperatures had a varied influence on bacterial growth. The nanocomposites synthesized at 25 °C (BW9Ag1 and BAg1) exhibited stronger inhibition of the bacterial strain than the nanocomposites synthesized at 80 °C (BW9Ag3 and BAg3) (Table 7).

**Figure 5:**
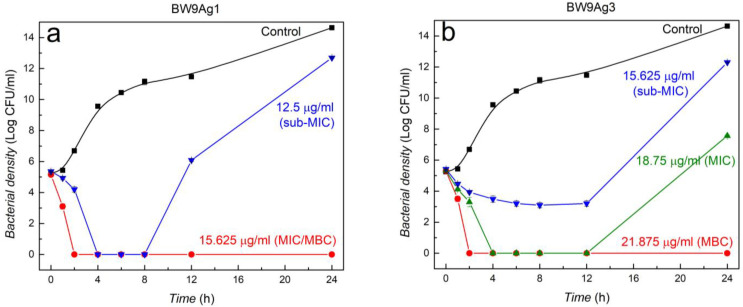

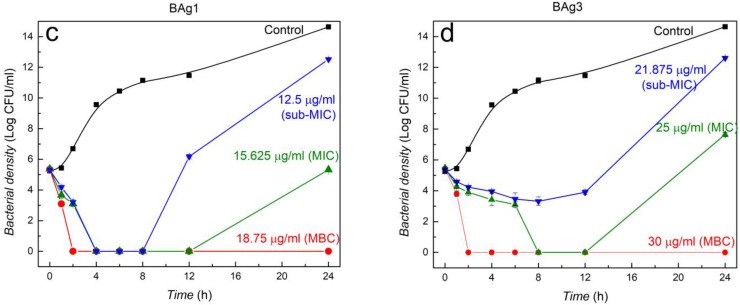
Time–kill curves for *E. coli* exposed to four different nanocomposites at different concentrations. Nanocomposites synthesized after 120 minutes’ reaction time using precursors BW9 at (**a**) 25 °C (BW9Ag1) and (**b**) 80 °C (BW9Ag3) and B at (**c**) 25 °C (BAg1) and (**d**) 80 °C (BAg3).

At MIC, nanocomposites inhibit bacterial growth until 12 h, then slight growth is seen between 12 and 24 h except for BW9Ag1 (Figure 5). However, at sub-MIC, the inhibition capacity of BW9Ag1 and BAg1 is adequate for 8 h, completely inhibiting the bacterial growth, while BW9Ag3 and BAg3 partially inhibit the growth until 12 h. After 12 h, the inhibition potency of nanocomposites stops, and bacteria regrows. The MIC of nanocomposites synthesized at 80 °C (BW9Ag3 and BAg3) is higher than nanocomposites synthesized at 25 °C (BW9Ag1 and BAg1), suggesting antimicrobial activity is inversely related to the synthesis temperature. Subsequently, it can be concluded that antimicrobial activity is strongly affected by Ag_3_PO_4_ and AgCl phases in the nanocomposites. The nanocomposites synthesized at 25 °C contain higher amounts of these phases than those synthesized at 80 °C and, therefore, exhibit better antibacterial activity. This observation ultimately suggests that Ag^+^ ions release is the primary factor responsible for the antibacterial activity of the nanocomposites.

**Figure 6:**
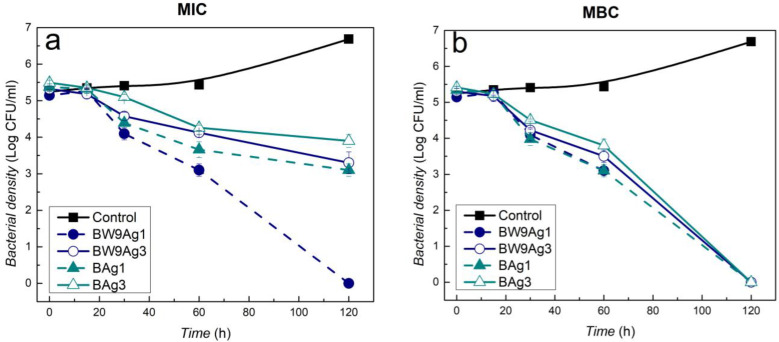
Time–kill kinetics for *E. coli* exposed to (**a**) MIC and (**b**) MBC concentrations of nanocomposites synthesized after 120 minutes’ reaction time using precursors BW9 and B at 25 °C (BW9Ag1 and BAg1) and 80 °C (BW9Ag3 and BAg3).

Time–kill kinetics depicts the rate at which the *E. coli* bacterial strain has been affected by the nanocomposites (Figure 6). At MIC, the nanocomposites synthesized at 25 °C (BW9Ag1 and BAg1) affect the *E. coli* bacterial strain faster than those synthesized at higher temperatures (BW9Ag3 and BAg3) (Figure 6a). At minimum bactericidal concentration (MBC), all the nanocomposites kill the bacteria after 2 h incubation (Figure 6b).

## 4. Discussion

Brewery waste from different stages and the final product have various organic content and minerals. The insoluble complexes formed through the reaction between polyphenols and soluble proteins are discarded at stages 5, 7, and 9 as they alter beer taste [60]. Therefore, the brewery waste from stage 5, BW5 [23], contains the highest amounts of these insoluble complexes, followed by BW7 [23] and BW9. Consequently, BW5 is rich in several compounds such as carbohydrates, reducing sugars, chlorine, and phosphorus. BW9, the last waste in the production line, contains the least amount of carbohydrates and reducing sugars, as they are already discarded in previous stages or consumed during the reaction.

The silver phosphate-containing nanocomposites obtained previously using brewery wastes BW5, and BW7 [23] will be included in this discussion to yield a global understanding of the nanocomposites’ formation and compositions and their subsequent effect on Gram-negative bacterial strain. Silver chloride nanoparticles were synthesized in major amounts using BW9 and B as precursors for nanoparticle synthesis. Synthesis time and temperature affected the incorporation of Ag_met_ in BW9 nanocomposites and Ag_met_ and Ag_3_PO_4_ nanoparticles in B nanocomposites in minor quantities. The reaction proceeds with the spontaneous formation of AgCl through a competing ion-exchange reaction which favors the exchange between AgNO_3_ and chlorine sources present in the waste. The remaining Ag^+^ ions, after AgCl nanoparticle formation, proceed through another ion-exchange reaction to produce Ag_3_PO_4_ nanoparticles if phosphates are available in an unbound form. BW9 produces only AgCl and Ag_met_ nanoparticles. Silver phosphate nanoparticles were not formed when BW9 was used as a synthesis agent despite containing significant phosphorus content. Three reasons can be postulated for Ag_3_PO_4_ not being produced by BW9: (a) the chlorine available for ion-exchange consumes Ag^+^ ions entirely, (b) the phosphorus present in BW9 is not available as unbound phosphate ions for ion-exchange, and (c) BW9 has the least carbohydrates and reducing sugars. With comparison from previous research [23], it can be observed that BW7, BW5, and B contain significant amounts of carbohydrates and sugars as opposed to BW9, which is relatively highly deficient. Therefore, it can be concluded that carbohydrates and sugars play an important role in the formation of silver phosphate.

Various chemicals present in plants like reducing sugars, carbohydrates, amides, and polyphenols are known to play a significant role in reducing silver ions to silver nanoparticles [61]. Oxidation of the aldehyde group to carboxylic group in sugars and carbohydrates occurs while reducing silver ions [62]. Polyphenols have antioxidant properties, oxidizing to Quinone and reducing silver ions to metallic silver [63,64]. The complexity of polyphenol and the total number of OH groups present determine the polyphenol’s antioxidant potential. The pH of the reaction mixture, the presence of O_2,_ and the reaction time affect the reduction of Ag^+^ ions by polyphenols [65]. The synthesis temperature of the reaction mixture also affects silver reduction as higher synthesis temperatures aid the silver ion reduction into silver nanoparticles.

In B nanocomposites, a small amount of Ag_met_ was formed even at 25 °C due to the presence of relatively higher reducing sugars, similar to BW5 nanocomposite previously reported [23]. BW9 yielded pure AgCl nanoparticles at 25 °C, and only increasing synthesis temperature aided the reduction of silver ions due to insignificant amounts of carbohydrates, reducing sugars and polyphenol content available for reduction. The crystallite size of AgCl and Ag_3_PO_4_ increases with synthesis temperature, indicating that the growth of nanoparticles is favored over nucleation. Concurrently, the crystallite size of Ag_met_ increases in BW9 nanocomposites but decreases in B nanocomposites as in BW5 nanocomposites [23]. Nucleation is favored over the growth of Ag_met_ nanoparticles by increasing synthesis temperature due to the higher reducing agents like carbohydrates, reducing sugars, and polyphenols content in B and BW5. The total Ag content in nanocomposites prepared using all precursors (BW9, B, BW5 [23], and BW7 [23]) slightly increases with increasing synthesis time and temperature. However, BW9 nanocomposites have the least Ag wt.% compared to those synthesized using B, BW5, and BW7, respectively, at the same synthesis conditions.

Comparing the elemental and chemical composition of BW9, B, BW5, and BW7 nanocomposites provides important information regarding their surface. As the synthesis temperature increases, the elemental composition of P increases for B nanocomposites but decreases for BW7 and BW5 nanocomposites. Although the Ag_3_PO_4_ content in BW5 nanocomposites increases with increasing synthesis temperature, the surface P content decreases, resulting in an overall decrease in P. The Cl wt.% decreases with increasing synthesis temperature for BW9, BW5, and B nanocomposites which could account for a decrease of AgCl and increase in Ag_met_ content in the composites. The surface composition of nanoparticles is affected by various reaction parameters, e.g., reagents, solvents, stabilizing agents, pH, synthesis temperature, and type of nanoparticles synthesized [23]. Reducing sugars and polyphenols contain carbonyl groups known to stabilize the nanoparticles [23]. The surface of nanocomposites with AgCl as a major phase shows a prominent presence of polyphenolic groups, C–O groups, and peptide-protein bindings on the surface. This could be due to these groups’ stronger affinity towards AgCl than Ag_3_PO_4_ nanoparticles. BW9 and B nanocomposites, similar to BW5 nanocomposites [23], have a higher surface carbon to oxygen ratio, attributed to oxygen-deficient moieties like polyphenols. Approximately 40–50% of the BW7 nanocomposites’ surface is composed of Ag_3_PO_4_, thus having higher silver and low carbon content (<25%). This suggests a relatively thinner organic coating (~17–25 Å) on BW7 nanocomposites. Due to this low carbon content on the surface, the carbon to oxygen ratio is lowest for BW7 nanocomposites. Therefore, BW9, B, and BW5 nanocomposites’ surfaces can be considered oxygen-deficient relative to the oxygen-enriched BW7 nanocomposites’ surface.

The nanocomposites exhibit antibacterial properties. The interaction of nanoparticles with microorganisms is complicated thus not clearly understood [66]. Some pathways through which silver nanoparticles will interact and affect the cells have been speculated through various research such as (a) nanoparticles could attach to the cell membrane and cause structural alterations and membrane damage, which eventually results in cell death; (b) diffusion of nanoparticles into the cell and damaging the cell organelles; (c) binding of Ag^+^ ions to sulfur and phosphorus sites of DNA, causing DNA damage and thus affecting the DNA replication; and (d) generation of reactive oxygen species (ROS) that can destroy cell organelles and receptors [67,68]. The size and surface of nanoparticles play an essential role in interaction with cells [69]. Smaller nanoparticles can bind strongly and have higher bactericidal potential than larger nanoparticles, owing to surface exposure to the bacterial cell wall [70].

The nanocomposites synthesized at temperatures of 25 and 80 °C exhibit high antimicrobial properties when tested on *E. coli*. The composites synthesized at 25 °C (BW9Ag1 and BAg1) inhibited bacterial growth more effectively than those synthesized at 80 °C (BW9Ag3 and BAg3), similar to that observed for BW5 and BW7 nanocomposites previously [23]. The higher surface content and smaller size of AgCl nanoparticles could be one of the reasons for the enhanced antimicrobial activity of nanocomposites synthesized at 25 °C in the case of nanocomposites (BW9, B, and BW5) with AgCl as the major phase. The nanocomposites synthesized at 80 °C have more silver oxides and Ag–O polymer groups on the surface, resulting in strong binding of silver to the surface, and the carbon overlayer is thick, thereby slowing the release of Ag^+^ into the environment. As previously reported [23], the higher solubility of Ag_3_PO_4_ than AgCl results in a higher amount of Ag^+^ ions for interaction with bacterial cells. However, the Ag_3_PO_4_ nanoparticles present in B nanocomposites do not form a part of the nanocomposite surface and, therefore, are ineffective in inhibiting microbial growth through Ag^+^ ion production and surface interactions with the bacteria. Therefore, the B nanocomposites exhibit the weaker antimicrobial activity in the set (BW9, B, BW5, BW7 nanocomposites). Therefore, it can be concluded from the current and previous study on Ag_3_PO_4_ containing silver nanocomposites that Ag_3_PO_4_ nanoparticles must be present on the surface to inhibit bacterial growth. Moreover, the surface carbon groups are crucial for attaching nanoparticles with the negatively charged cell wall of Gram-negative *E. coli* [71]. These carbon groups observed in XPS analysis (Csp^3^, C = O, Csp^2^, and C–OH) indicate the presence of sugars, carbohydrates, polyphenols, and other organic compounds on the nanoparticle surface. Surfaces rich in oxygen that are not C-bonded have exhibited higher antimicrobial activity; consequently, BW9 and B nanocomposites exhibit slightly lower antimicrobial activity than BW7 and BW5 nanocomposites from a previous study [23]. This indicates that the surface O content and chemical form play a significant role in inhibition capacity/antibacterial property. In particular, although the surface of the B nanocomposites is richer in O content than BW9 nanocomposites, they exhibit lower antibacterial activity because of higher C bonded oxygen content. Moreover, the BW9 and B nanocomposites synthesized at 25 °C, exhibiting relatively better antibacterial activity, also contain fewer Csp^2^ and C–OH groups on the surface. Previously studied BW7 and BW5 nanocomposites [23] have demonstrated slightly better antimicrobial activity than currently studied BW9 and B nanocomposites. The antibacterial activity can be predominantly attributed to the role of Ag^+^ ions release and the adsorption properties of the hydrolyzed oxygen groups present on the nanocomposites’ surface, which lead to biological material damage. The surface Ag_3_PO_4_ and oxygen content are higher for BW7 and BW5 nanocomposites, which play a significant role in improving the inhibition capacity of the nanocomposites. Moreover, the chemical forms of the surface moieties and the thickness of the capping also affected the antimicrobial activity. Therefore, this leads us to conclude that these are the major factors that influence the antimicrobial activity of the nanocomposites.

Brewery by-product (BW9) and the final product (B) were valorized successfully to synthesize silver chloride nanocomposites containing silver and silver phosphate via a simple, fast, and cost-effective method that is highly reproducible. These nanocomposites can be applied as antimicrobial agents for disinfecting air, water, and contaminated surfaces specifically to treat hospital-acquired infections. Further biocompatibility assessment is required to enable advanced clinical applications.

BW9 has not been investigated in detail previously in the literature for its composition among the brewery wastes. BW9 consists of significantly lower nutrients than other brewery wastes dissolved in high amounts of water, so this waste has not been actively studied. However, considering the large amount of this waste produced during the brewing process, it is crucial to study the composition of this BW9 waste and find alternative recycling options for it. In our previous work [23], wort precipitate BW5 and Brewer’s spent yeast BW7 have been reported, which yielded nanocomposites consisting of high amounts of Ag_3_PO_4_ in the structure along with AgCl and Ag_met_. Disparately, herein, BW9 has been used for obtaining pure AgCl and nanocomposites of AgCl, including Ag_met_ in the structure. BW9 being the last stage of waste in beer production, the nanocomposites synthesized using BW9 have been compared with those synthesized using the brewery product B.

## 5. Conclusions

- Nanocomposites with AgCl as the major phase and Ag metal were synthesized using BW9 and B. Additionally, the Ag_3_PO_4_ phase was also present in B nanocomposites.
- Increasing synthesis temperature aided the growth of minor phases into the composite.
- The nanocomposites’ surface is composed of organic groups present in the brewery products BW9 and B. The resulting overlayer thickness was ~23–30 Å. The surface of B nanocomposites is enriched in oxygen compared to BW9 nanocomposites.
- Nanocomposites showed antibacterial activity towards Gram-negative strain *Escherichia coli* (*E. coli* ATCC 25922).
- Better antibacterial activity was exhibited by BW9 nanocomposite due to the larger AgCl, Ag oxides content, and smaller content of C–OH and C = O groups on the surface than in B nanocomposite.

## Figures and Tables

**Table 1:** Composition analysis of BW9 and B.

Analysis	Subtype	Units	Brewery Wastes	
			BW9	B
Total nitrogen		mg L^−1^	443.8	782.6
Total polyphenols			87.75	213.65
Total sulfates			103.4	121.1
Total carbohydrates			1200	38,500
Fermentable sugars	Total		600	22,800
	Fructose		100	300
	Glucose		100	16,700
	Maltose + sucrose		200	1500
	Maltotriose		200	4300
Elemental content	K	mg L^−1^	284 ± 48.3	558 ± 94.9
	P		149 ± 6	266 ± 10.6
	Cl		165 ± 1.0	286 ± 2.0
	Mg		56.1 ± 9.5	103.9 ± 17.7
	Ca		68.0 ± 10.2	27.8 ± 4.2
	Na		42.9 ± 4.3	55.8 ± 5.6
	Mn		0.22 ± 0.02	0.14 ± 0.01
	Fe		6.0 ± 0.84	0.2 ± 0.03
	Al		0.01 ± 0.01	0.09 ± 0.01
	Ni		<0.05	<0.05
	Zn		<0.05	<0.05
	Cu		<0.05	<0.05
Total organic carbon		mg L^−1^	2.84	6.43

**Table 2:** Phase content and nanocrystallites size of BW9 and B nanocomposites.

Sample	Synthesis Temperature (°C)	Synthesis Time (min)	PXRD Analysis					
			Ag Phase Content (wt.%)			Nanocrystallites Size (nm)		
			Ag_3_PO_4_	AgCl	Ag_met_	Ag_3_PO_4_	AgCl	Ag_met_
BW9Ag1	25	120		100	-		5.6 ± 0.5	-
BW9Ag2	50			97.0	3.0		5.6 ± 0.6	2.9
BW9Ag3	80			73.2	26.8		19.4 ± 3.1	3.6
BW9Ag4	80	30		75.7	24.3		15.6 ± 2.7	3.2
BW9Ag5	80	10		92.9	7.1		16.0 ± 2.7	2.5
BAg1	25	120	4.2	94.2	1.6	4.9	6.1 ± 1	10.9
BAg2	50		2.0	96.7	1.3	9.2	7.9 ± 1.7	7.6
BAg3	80		19.1	51.8	29.1	10.8 ± 5.2	10.7 ± 1.2	3.0 ± 0.3
BAg4	80	30	15.8	61.5	22.7	11 ± 5.5	10.4 ± 2.4	3.3
BAg5	80	10	33.4	63.8	2.8	9.8 ± 3.9	9.8 ± 1.8	10.1

**Table 3:** Elemental composition of BW9 and B nanocomposites.

Sample	Synthesis Temperature (°C)	Synthesis Time (min)	XRF Analysis			
			Elemental Composition (wt.%)			
			Ag	P	Cl	S
BW9Ag1	25	120	83.1	0.1	15.1	1.6
BW9Ag2	50		83.8	0.2	14.6	1.5
BW9Ag3	80		89.3	0.6	9.3	0.8
BW9Ag4	80	30	88.0	0.4	10.7	0.9
BW9Ag5	80	10	84.4	0.3	14.5	0.8
Bag1	25	120	86.4	0.5	12.0	1.1
Bag2	50		85.3	0.7	13.0	1.1
Bag3	80		91.9	1.4	6.1	0.6
Bag4	80	30	91.1	1.3	7	0.6
Bag5	80	10	91.1	2.6	5.8	0.5

**Table 4:** Surface weight composition of BW9 and B nanocomposites obtained from XPS analysis.

Sample	Synthesis Temperature (°C)	Synthesis Time (min)	XPS Analysis							
			Surface Elemental Composition (wt.%)							
			C	O	Ag	N	P	S	B	Cl
BW9Ag1	25	120	35.2	10.5	38.3	5.1	-	1.0	1.2	8.7
BW9Ag2	50		41.4	13.8	31.4	5.4		1.1	0.7	6.2
BW9Ag3	80		36.8	13.3	38.3	5.8		1.3	-	4.5
BW9Ag4	80	30	37.7	12.3	39.8	5.6		0.5		4.1
BW9Ag5	80	10	36.1	12.1	38.8	5.2		1.5		6.3
BAg1	25	120	33.8	14.1	39.4	5.4	0.4	0.9	0.7	5.3
BAg2	50		29.8	13.0	39.3	5.8	0.6	1.2	5.3	5.0
BAg3	80		26.5	13.7	51.1	5.0	0.6	0.8	-	2.3
BAg4	80	30	26.5	12.8	49.6	4.3	1.9	1.7		3.2
BAg5	80	10	33.3	16.6	38.8	6.5	1.6	0.4		2.8

**Table 5:** Surface Ag chemical state content in BW9 and B nanocomposites obtained from XPS analysis.

Sample	Synthesis Temperature (°C)	Synthesis Time (min)	Ag Chemical State (wt.%)—Ag 3d_5/2–3/2_			
			AgO-Polymer	Ag_2_O, AgO, AgOH	Ag_3_PO_4_	AgCl
			365.4 ± 0.3 eV (BW9) 364.9 ± 0.4 eV (B)	BE = 367.3 eV	BE = 367.8 eV	BE = 368.1 eV
BW9Ag1	25	120	5.01	1.75	-	31.54
BW9Ag2	50		3.90	1.20	-	26.30
BW9Ag3	80		7.19	2.96	-	28.15
BW9Ag4	80	30	6.45	3.52	-	29.83
BW9Ag5	80	10	6.03	2.17	-	30.60
BAg1	25	120	3.38	0.38	-	35.74
BAg2	50		3.19	0.75	-	36.96
BAg3	80		4.36	-	-	35.64
BAg4	80	30	4.79	-	-	37.11
BAg5	80	10	3.96	-	4.33	38.81

**Table 6:** Surface C chemical state content in BW9 and B nanocomposites obtained from XPS analysis.

Sample	Synthesis Temperature (°C)	Synthesis Time (min)	C Chemical State (wt.%)—C 1s				XPS QUASES-TougaardBL Model Overlayer Thickness (Å)
			C sp^2^	C sp^3^	C–OH	C=O	
			BE = 284.4 ± 0.1 eV	BE = 285.3 ± 0.1 eV	BE = 286.2 ± 0.1 eV	BE = 287.3 ± 0.1 eV	
BW9Ag1	25	120	17.31	12.13	-	5.76	23.5
BW9Ag2	50		22.75	12.40	1.56	4.69	28.9
BW9Ag3	80		20.68	6.88	6.88	2.36	27.7
BW9Ag4	80	30	21.93	7.19	5.83	2.75	27.3
BW9Ag5	80	10	17.75	11.40	4.37	2.58	28.0
BAg1	25	120	9.06	16.31	0.88	7.55	25.9
BAg2	50		8.74	13.34	1.97	5.75	24.5
BAg3	80		11.81	35.16	2.17	12.06	26.5
BAg4	80	30	9.04	38.61	-	11.75	24.3
BAg5	80	10	6.69	15.77	-	6.24	24.3

**Table 7:** Minimum inhibitory concentration (MIC) of BW9 and B nanocomposites for *E. coli*.

Sample	Synthesis Temperature (°C)	Synthesis Time (min)	MIC (μg mL^−1^)
			*Escherichia coli*
BW9Ag1	25	120	15.625
BW9Ag3	80		18.75
BAg1	25		15.625
BAg3	80		25

## Data Availability

The data are available in the Research group 18 data storage systems at the Institute of Physical Chemistry, Polish Academy of Sciences, Warsaw, Poland.

## Supplementary Materials

The following are available online at https://www.mdpi.com/article/10.3390/nano12030442/s1, Figure S1a: Elemental mapping of BW9Ag1 nanocomposite. Figure S1b: Elemental mapping of BW9Ag3 nanocomposite. Figure S1c: Elemental mapping of BAg1 nanocomposite. Figure S1d: Elemental mapping of BAg3 nanocomposite. Figure S2: The XPS survey spectra of BW9 and B nanocomposites synthesized at different temperatures and times. Figure S3a: C to Ag weight ratio variation for BW9 and B nanocomposites with synthesis (a) temperature and (b) time at 80 °C. Figure S3b: C to O weight ratio variation for BW9 and B nanocomposites with synthesis (a) temperature and (b) time at 80 °C. Figure S3c: Ag to O weight ratio variation for BW9 and B nanocomposites with synthesis (a) temperature and (b) time at 80 °C. Figure S4: Elementary weight composition comparison resulting from EDXRF and XPS spectra of nanomaterials synthesized at different temperatures and times using (a,c) brewery waste BW9 and (b,d) product B. Figure S5a: The Gaussian–Lorentzian asymmetric functions to different atomic chemical states fitted Ag 3d_5/2–3/2_ XPS spectra recorded from BW9 and B nanocomposites synthesized at different temperatures and times. Figure S5b: The Gaussian–Lorentzian asymmetric functions to different atomic chemical states fitted C 1s XPS spectra recorded from BW9 and B nanocomposites synthesized at different temperatures and times. Figure S5c: The Gaussian–Lorentzian asymmetric functions to different atomic chemical states fitted O 1s XPS spectra recorded from BW9 and B nanocomposites synthesized at different temperatures and times. Figure S6: Weight and normalized phase content comparison resulting from XRD and XPS spectra, respectively, in nanocomposites synthesized at different temperatures and times using (a,c) brewery waste BW9 and (b,d) product B. Figure S7a: QUASES-Analyze software and Buried Layer (BL) model analysis of Ag 3d_5/2/3-2_ spectra for BW9 nanomaterials at different synthesis temperatures and times at 80 °C. Figure S7b: QUASES-Analyze software and Buried Layer (BL) model analysis of Ag 3d_5/2/3-2_ spectra for B nanomaterials at different synthesis temperatures and times at 80 °C.

## Patent

The Polish provisional patent (P.433717/PK7364KZ) has been applied by the Institute of Physical Chemistry of the Polish Academy of Sciences.
