# Supplementary material for: Valorizing the Unexplored Filtration Waste of Brewing Industry for Green Silver Nanocomposite Synthesis
Source: Nanomaterials (Basel). 2022 Jan 28;12(3):442. doi: 10.3390/nano12030442 (PMC8839514; doi:10.3390/nano12030442)
Supplement: Supplementary file 1 [file nanomaterials-12-00442-s001.zip › nanomaterials-1500514-proof-check-SI/nanomaterials-1500514-proof-check-SI (1).pdf]

Valorizing the Unexplored Filtration Waste of Brewing Industry for Green Silver Nanocomposite Synthesis

Neha Venkatesh Rangam [1,*,†], Alcina Johnson Sudagar [1,*,†], Artur Ruszczak [1], Paweł Borowicz [1], József Tóth [2], László Kövér [2], Dorota Michałowska [3], Marek Łukasz Roszko [3], Krzysztof R. Noworyta [1] and Beata Lesiak [1]

[1] Institute of Physical Chemistry, Polish Academy of Sciences, Kasprzaka 44/52, 01-224 Warsaw, Poland; aruszczak@ichf.edu.pl (A.R.); pborowicz@ichf.edu.pl (P.B.); knoworyta@ichf.edu.pl (K.R.N.); blesiak-orlowska@ichf.edu.pl (B.L.)

[2] Institute for Nuclear Research, BemTér 18/c, H-4026 Debrecen, Hungary; toth.jozsef@atomki.hu (J.T.); kover.laszlo@atomki.hu (L.K.)

[3] Institute of Agriculture and Food Biotechnology—State Research Institute, ul. Rakowiecka 36, 02-532 Warsaw, Poland; dorota.michalowska@ibprs.pl (D.M.); marek.roszko@ibprs.pl (M.Ł.R.)

* Correspondence: nrangam@ichf.edu.pl or nehavr25@gmail.com (N.V.R.); asudagar@ichf.edu.pl or sudagaralcinajohnson@gmail.com (A.J.S.)

† These authors contributed equally to this work.

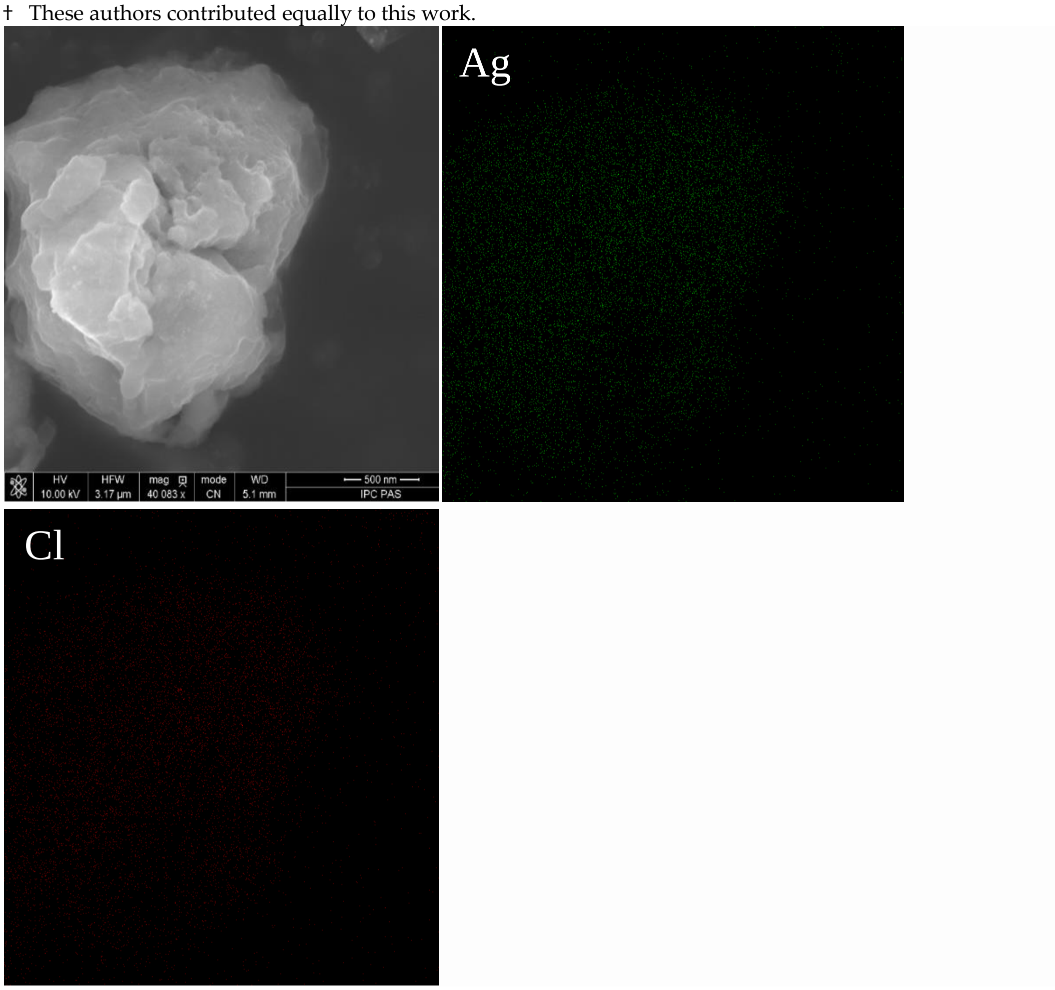

Figure S1a. Elemental mapping of BW9Ag1 nanocomposite.

Citation: Rangam, N.V.; Sudagar, A.J.; Ruszczak, A.; Borowicz, P.; Tóth, J.; Kövér, L.; Michałowska, D.; Roszko, M.Ł.; Noworyta, K.R.; Lesiak, B. Valorizing the Unexplored Filtration Waste of Brewing Industry for Green Silver Nanocomposite Synthesis. *Nanomaterials* **2022**, *12*, 442. https://doi.org/10.3390/nano12030442

Academic Editor(s): Francisco Alonso

Received: 24 November 2021
Accepted: 26 January 2022
Published: 28 January 2022

Publisher's Note: MDPI stays neutral with regard to jurisdictional claims in published maps and institutional affiliations.

Copyright: © 2022 by the authors. Submitted for possible open access publication under the terms and conditions of the Creative Commons Attribution (CC BY) license (https://creativecommons.org/licenses/by/4.0/).

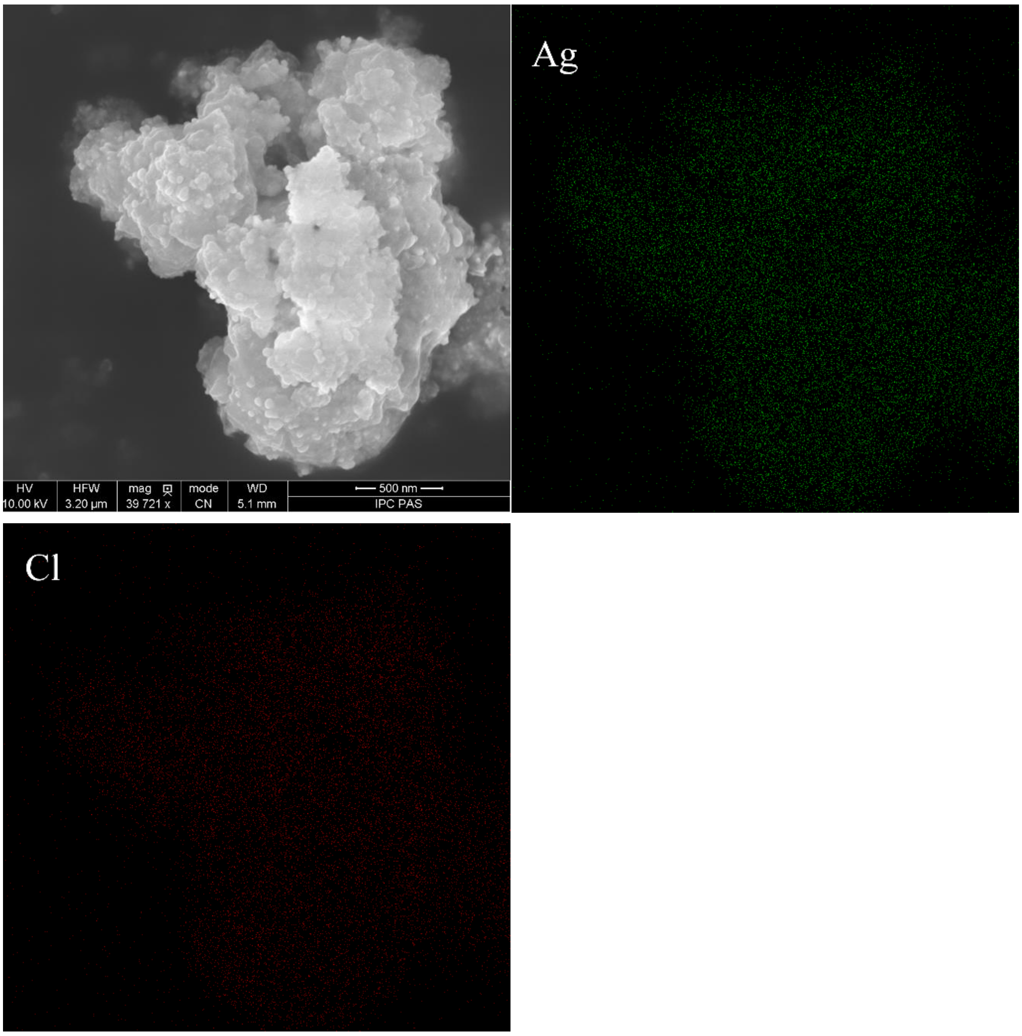

Figure S1b. Elemental mapping of BW9Ag3 nanocomposite.

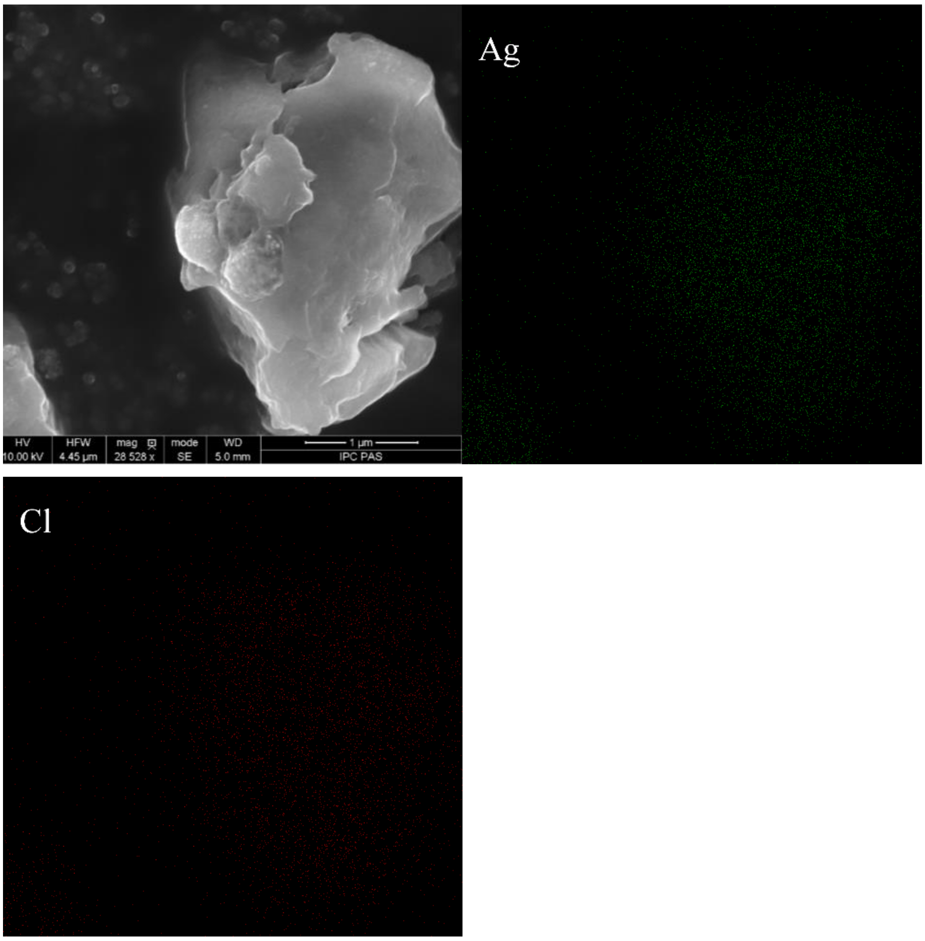

Figure S1c. Elemental mapping of BAg1 nanocomposite.

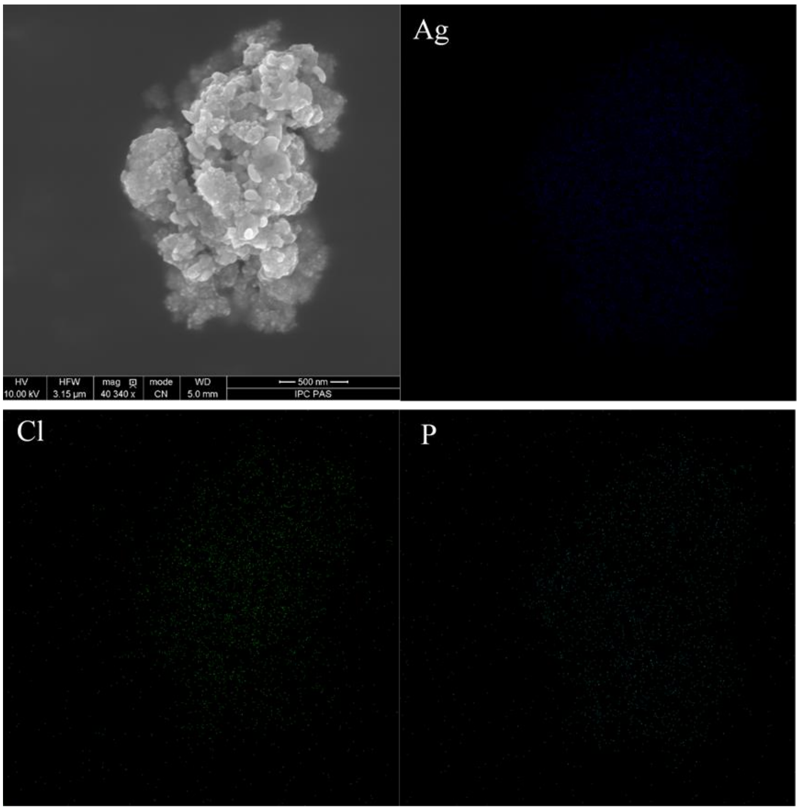

Figure S1d. Elemental mapping of BAg3 nanocomposite.

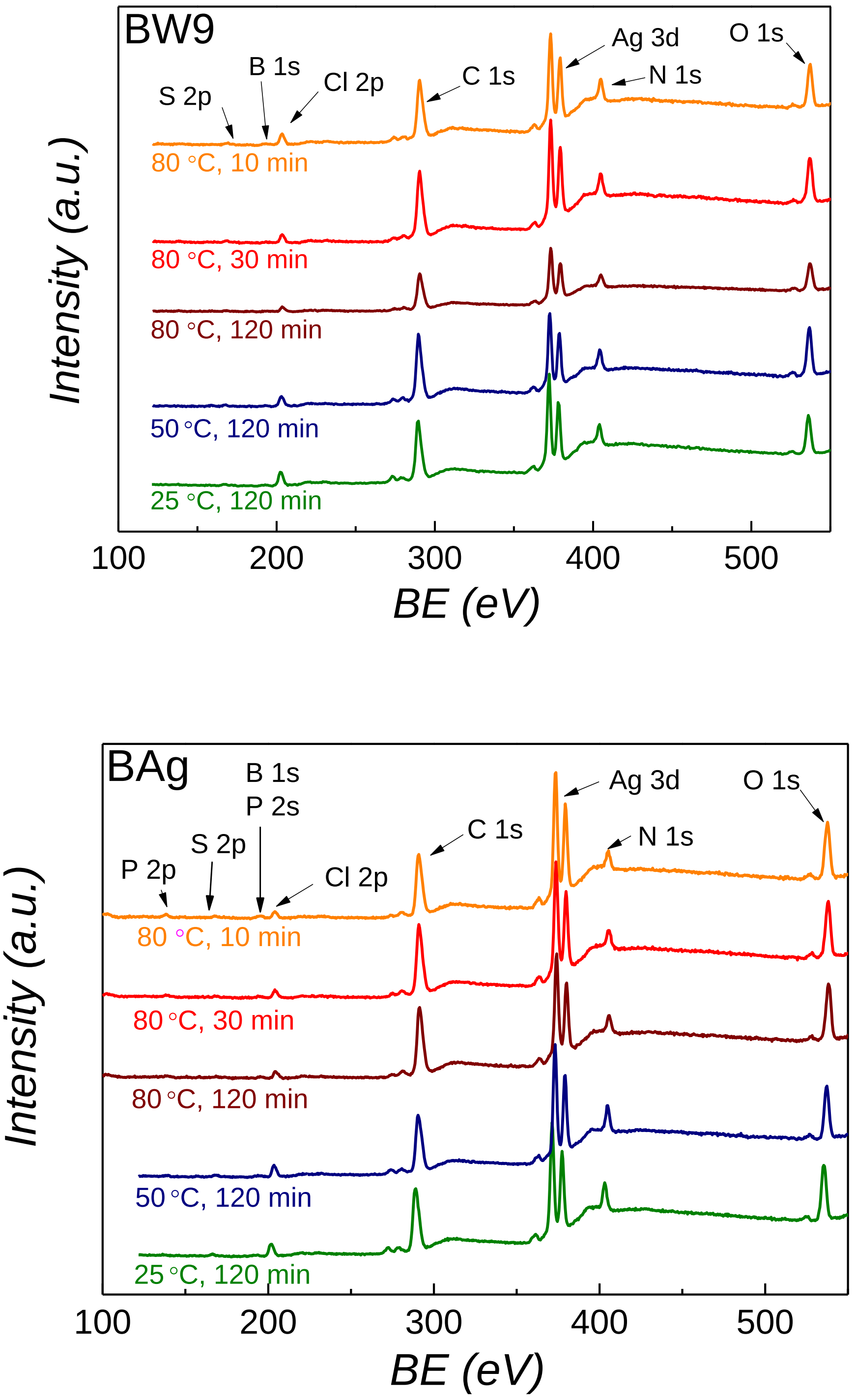

Figure S2. The XPS survey spectra of BW9 and B nanocomposites synthesized at different temperatures and times.

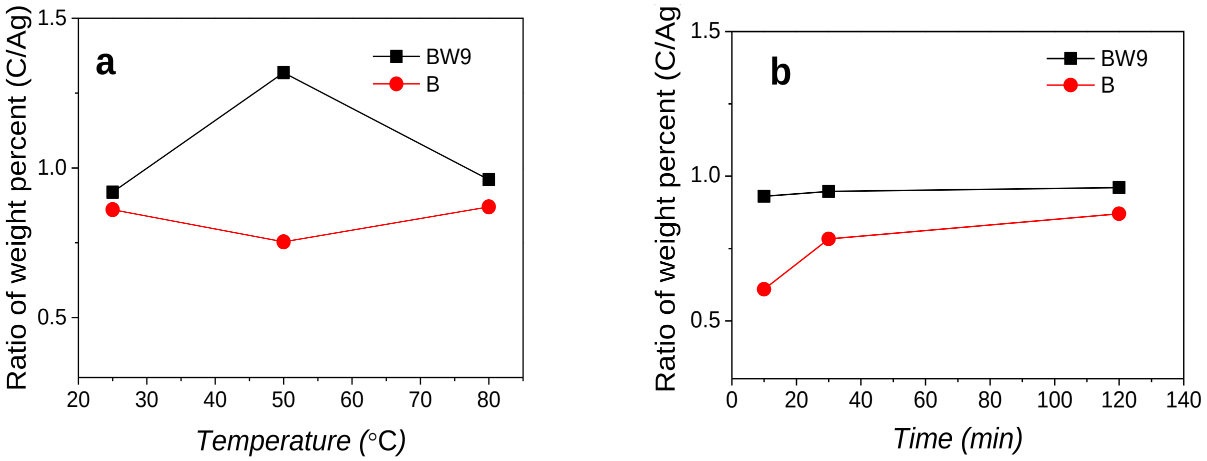

Figure S3a. C to Ag weight ratio variation for BW9 and B nanocomposites with synthesis (a) temperature and (b) time at 80 °C.

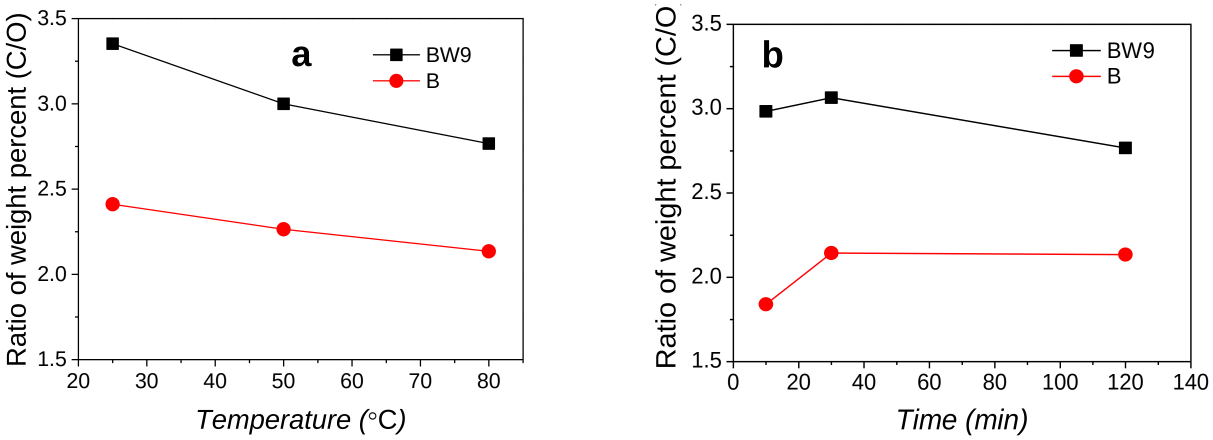

Figure S3b. C to O weight ratio variation for BW9 and B nanocomposites with synthesis (a) temperature and (b) time at 80 °C.

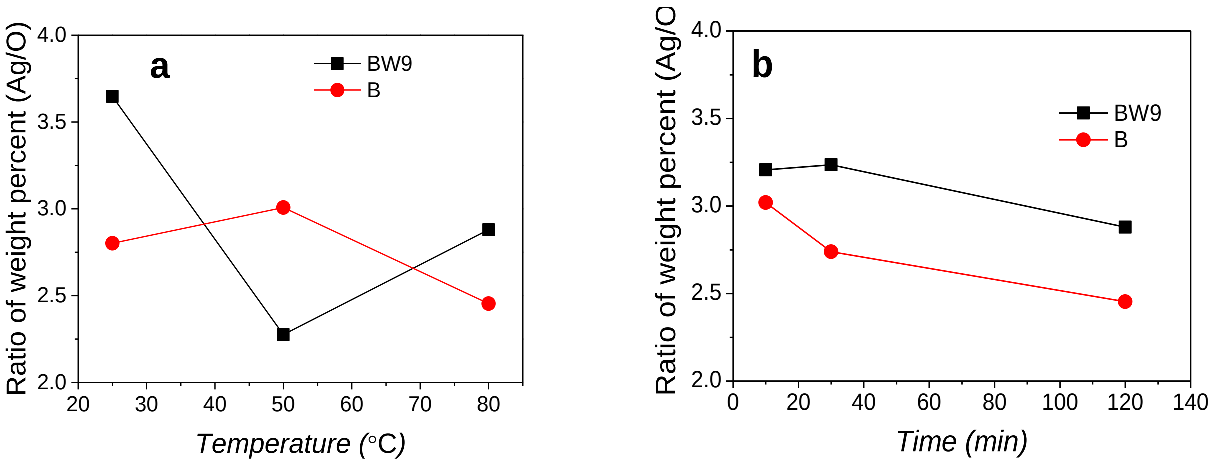

Figure S3c. Ag to O weight ratio variation for BW9 and B nanocomposites with synthesis (a) temperature and (b) time at 80 °C.

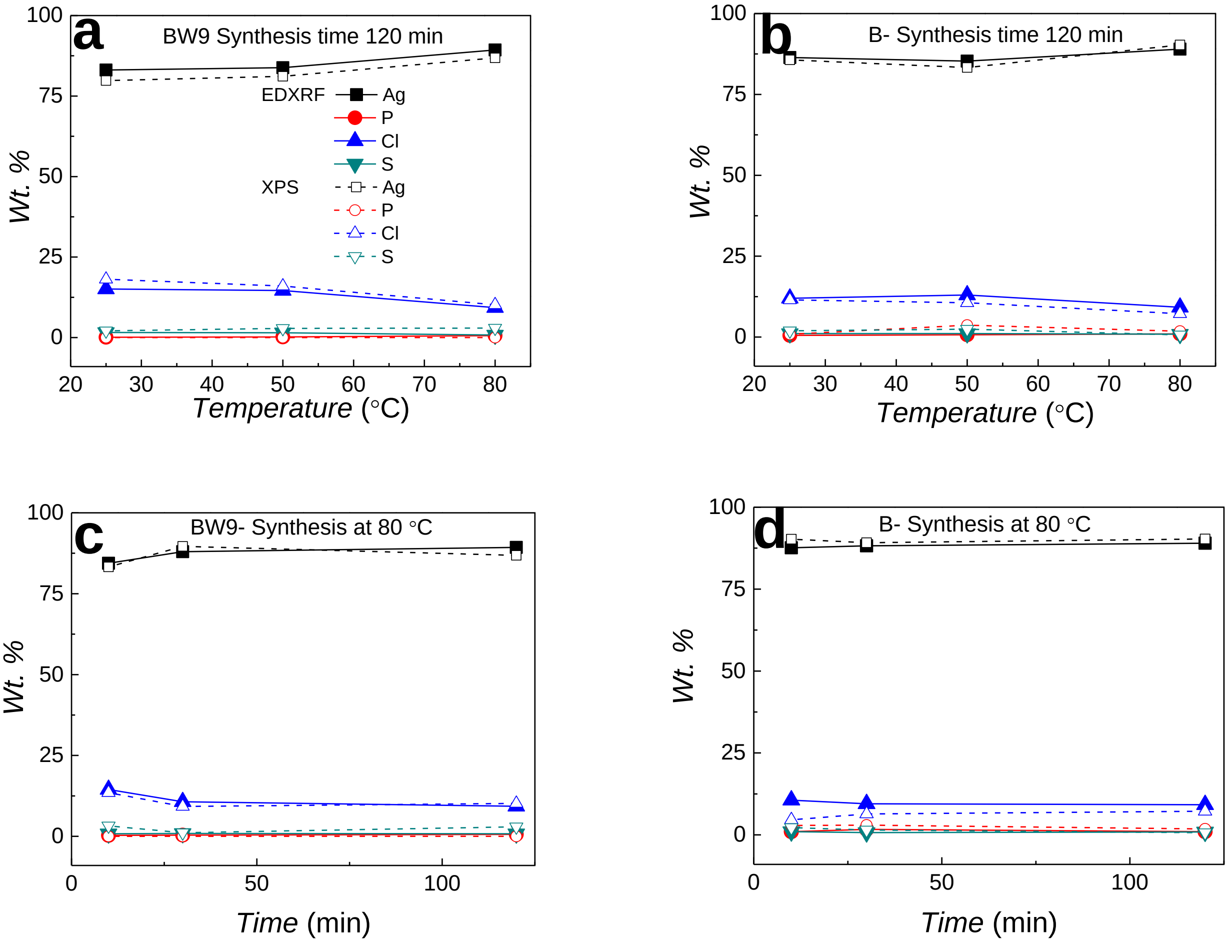

Figure S4. Elementary weight composition comparison resulting from EDXRF and XPS spectra of nanomaterials synthesized at different temperatures and times using (**a**,**c**) brewery waste BW9 and (**b**,**d**) product B.

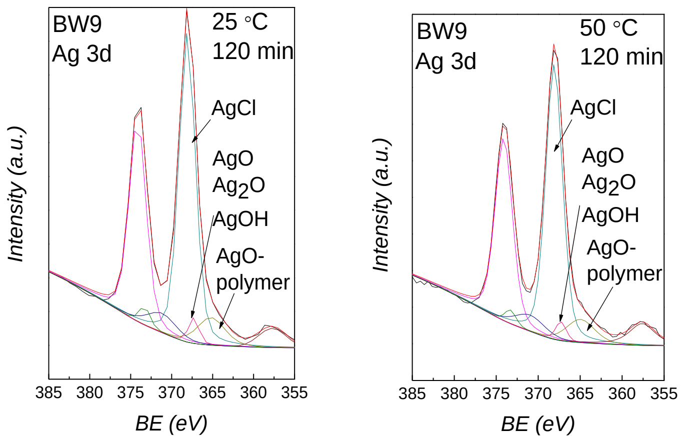

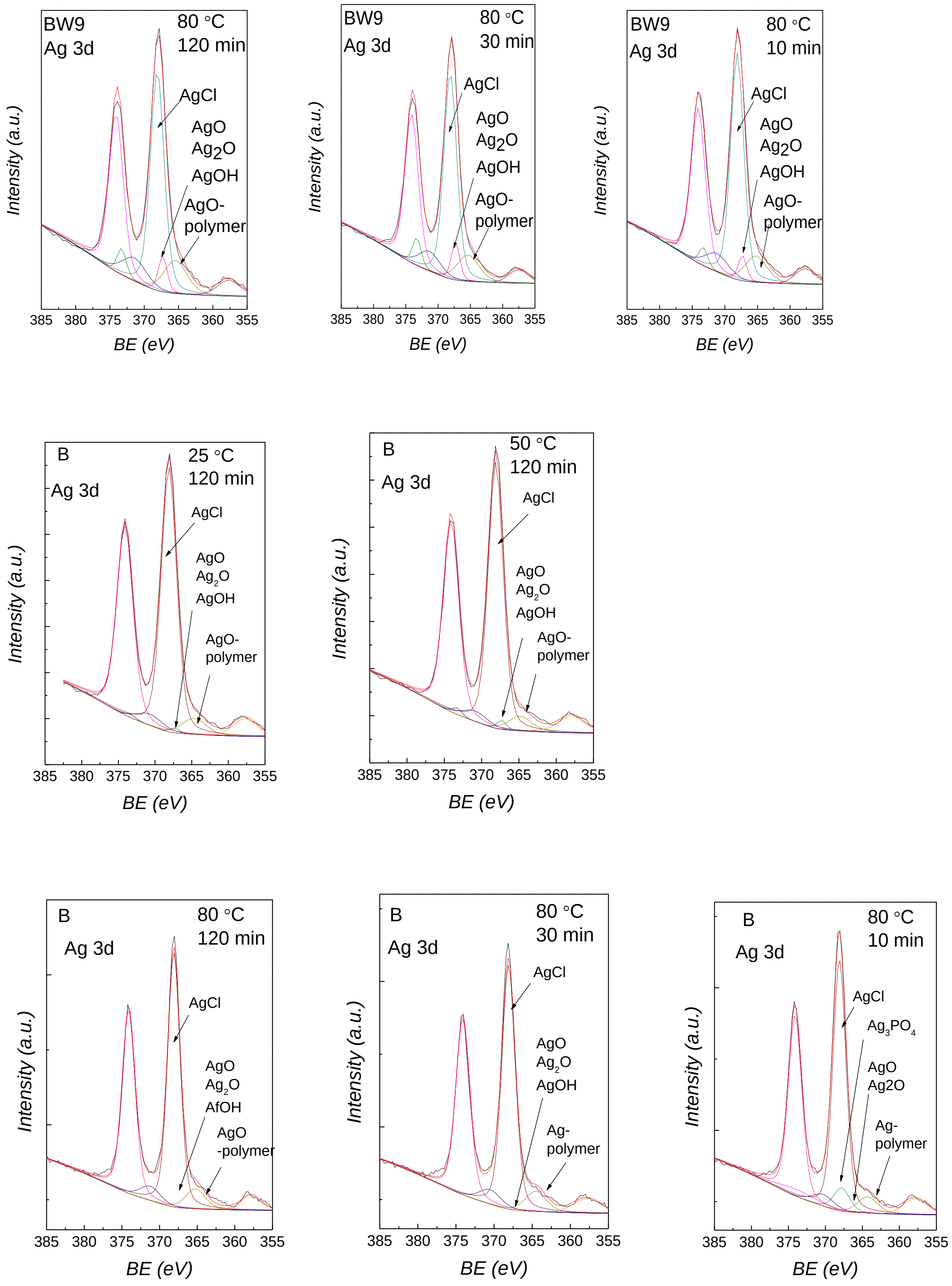

Figure S5a. The Gaussian–Lorentzian asymmetric functions to different atomic chemical states fitted Ag $3d_{5/2–3/2}$ XPS spectra recorded from BW9 and B nanocomposites synthesized at different temperatures and times.

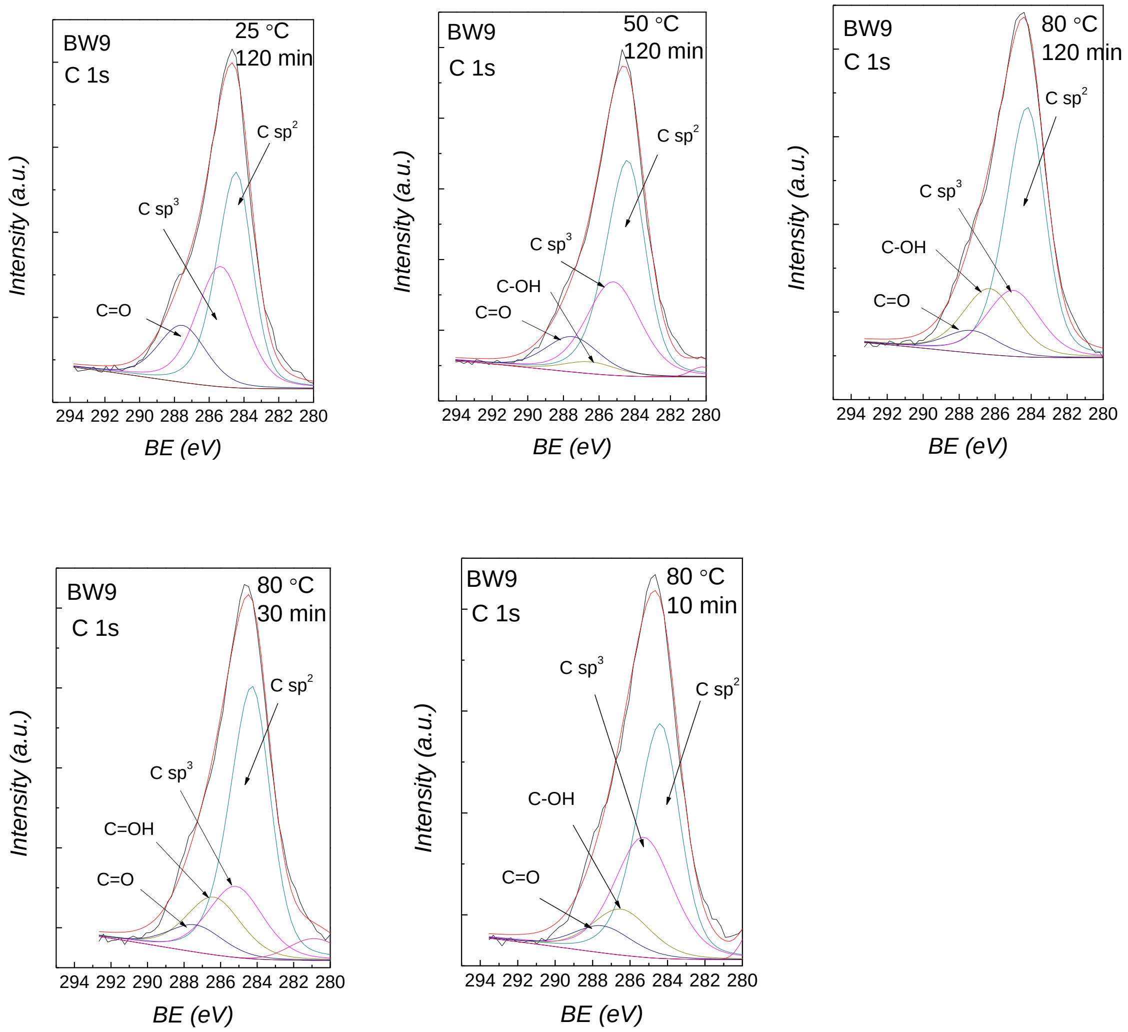

BW9
C 1s
25 °C
120 min
C sp2
C sp3
C=O
Intensity (a.u.)
294 292 290 288 286 284 282 280
BE (eV)
BW9
C 1s
50 °C
120 min
C sp2
C sp3
C-OH
C=O
Intensity (a.u.)
294 292 290 288 286 284 282 280
BE (eV)
BW9
C 1s
80 °C
120 min
C sp2
C sp3
C-OH
C=O
Intensity (a.u.)
294 292 290 288 286 284 282 280
BE (eV)
BW9
C 1s
80 °C
30 min
C sp2
C sp3
C=OH
C=O
Intensity (a.u.)
294 292 290 288 286 284 282 280
BE (eV)
BW9
C 1s
80 °C
10 min
C sp3
C sp2
C-OH
C=O
Intensity (a.u.)
294 292 290 288 286 284 282 280
BE (eV)

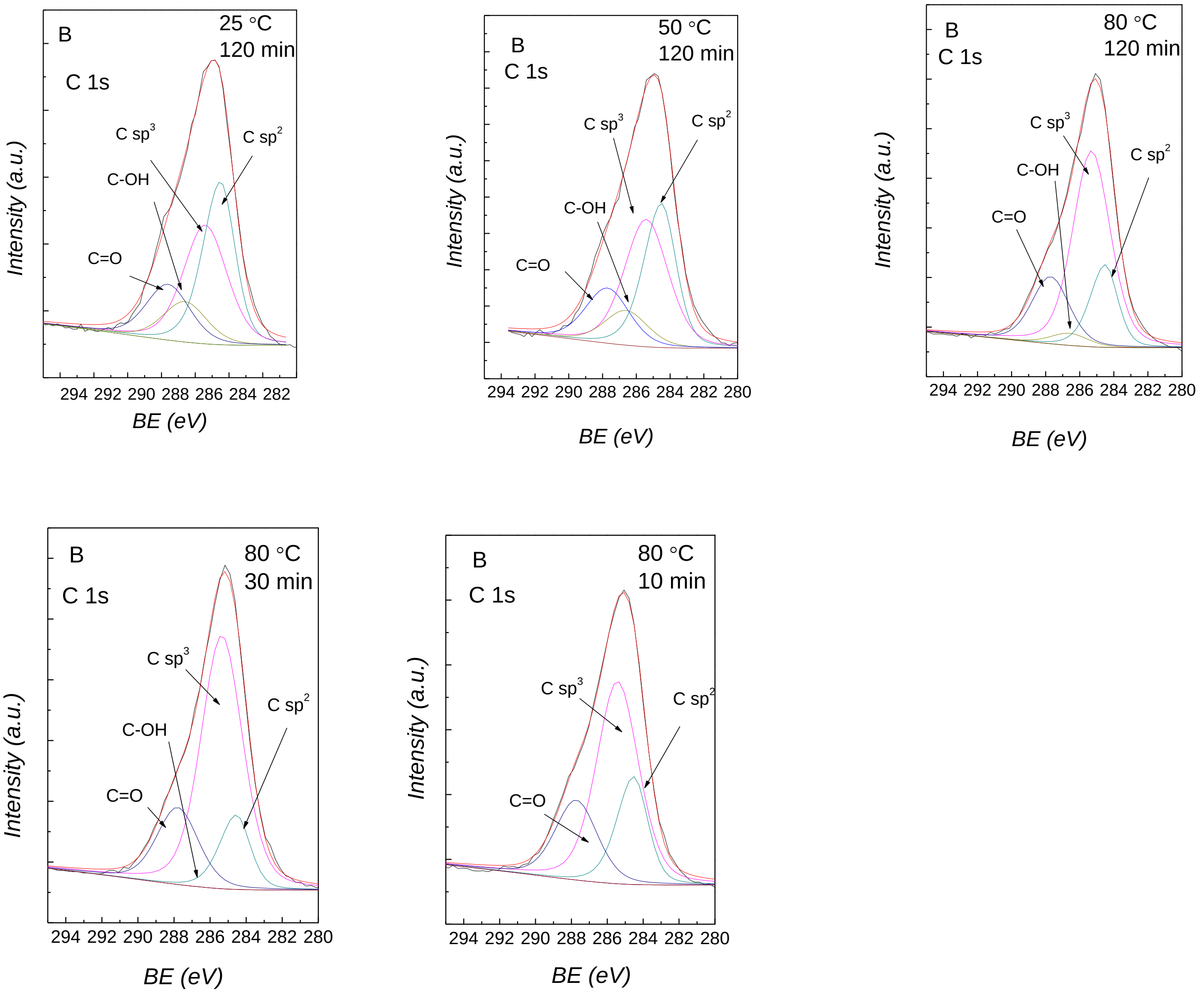

Figure S5b. The Gaussian–Lorentzian asymmetric functions to different atomic chemical states fitted C 1s XPS spectra recorded from BW9 and B nanocomposites synthesized at different temperatures and times.

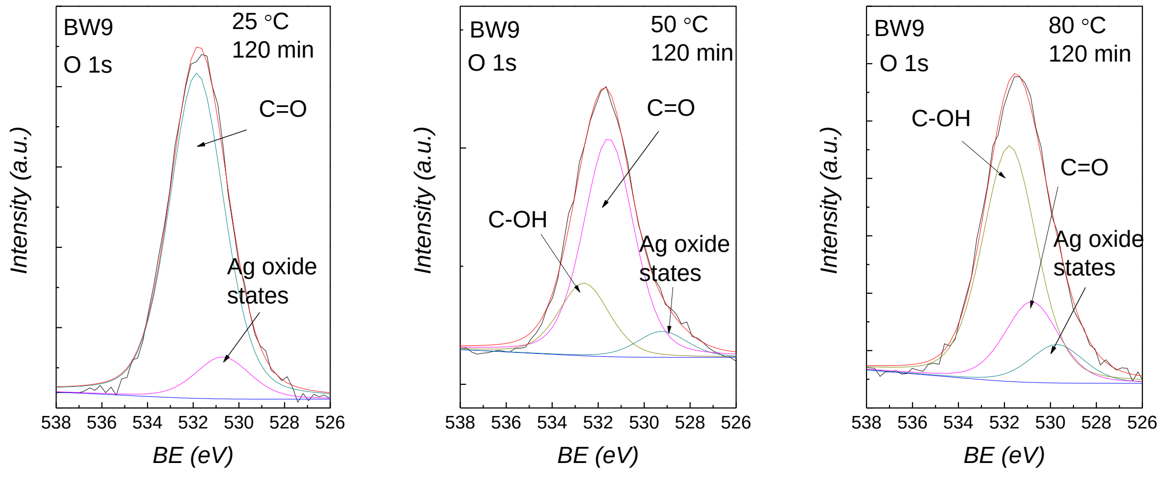

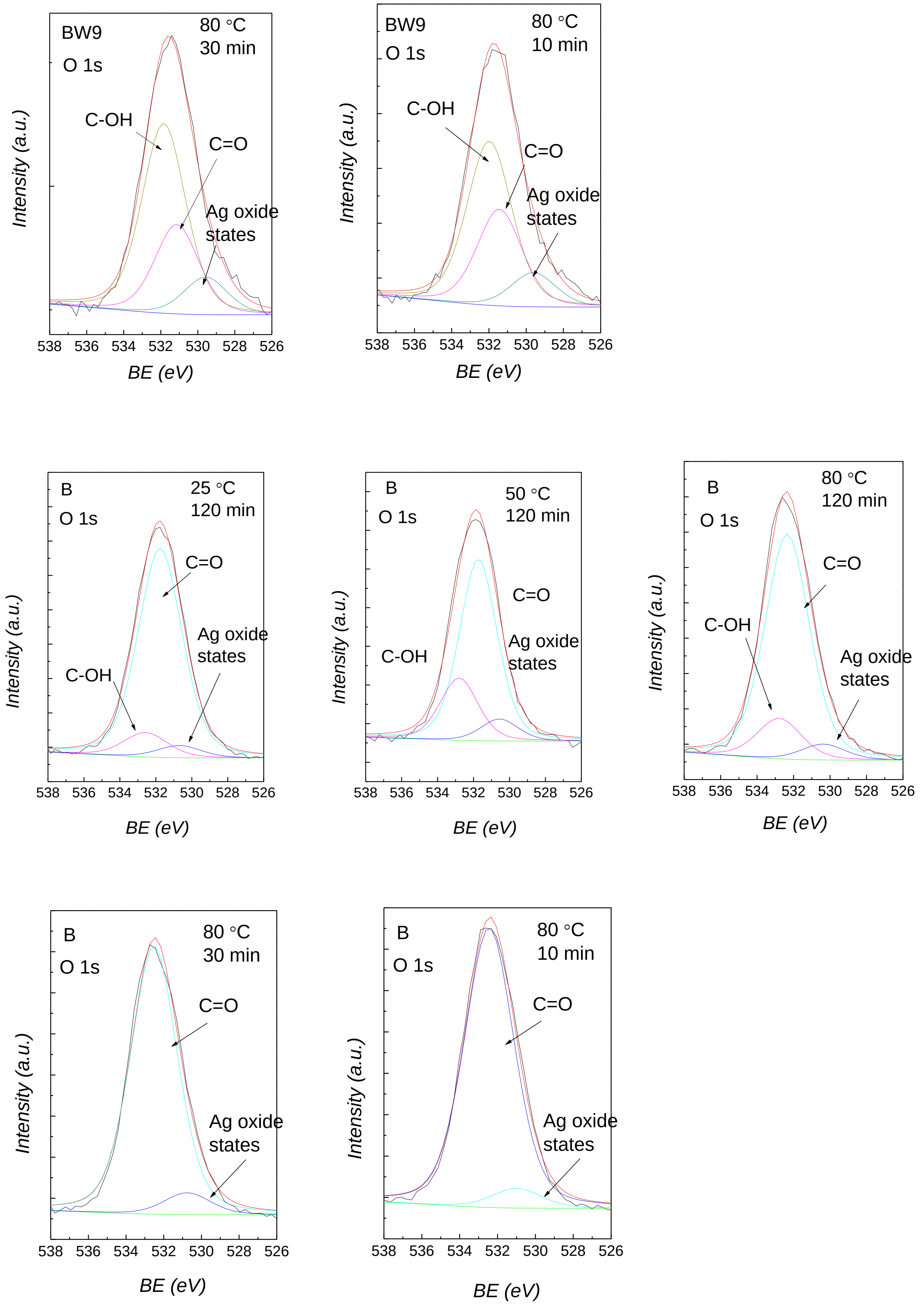

Figure S5c. The Gaussian–Lorentzian asymmetric functions to different atomic chemical states fitted O 1s XPS spectra recorded from BW9 and B nanocomposites synthesized at different temperatures and times.

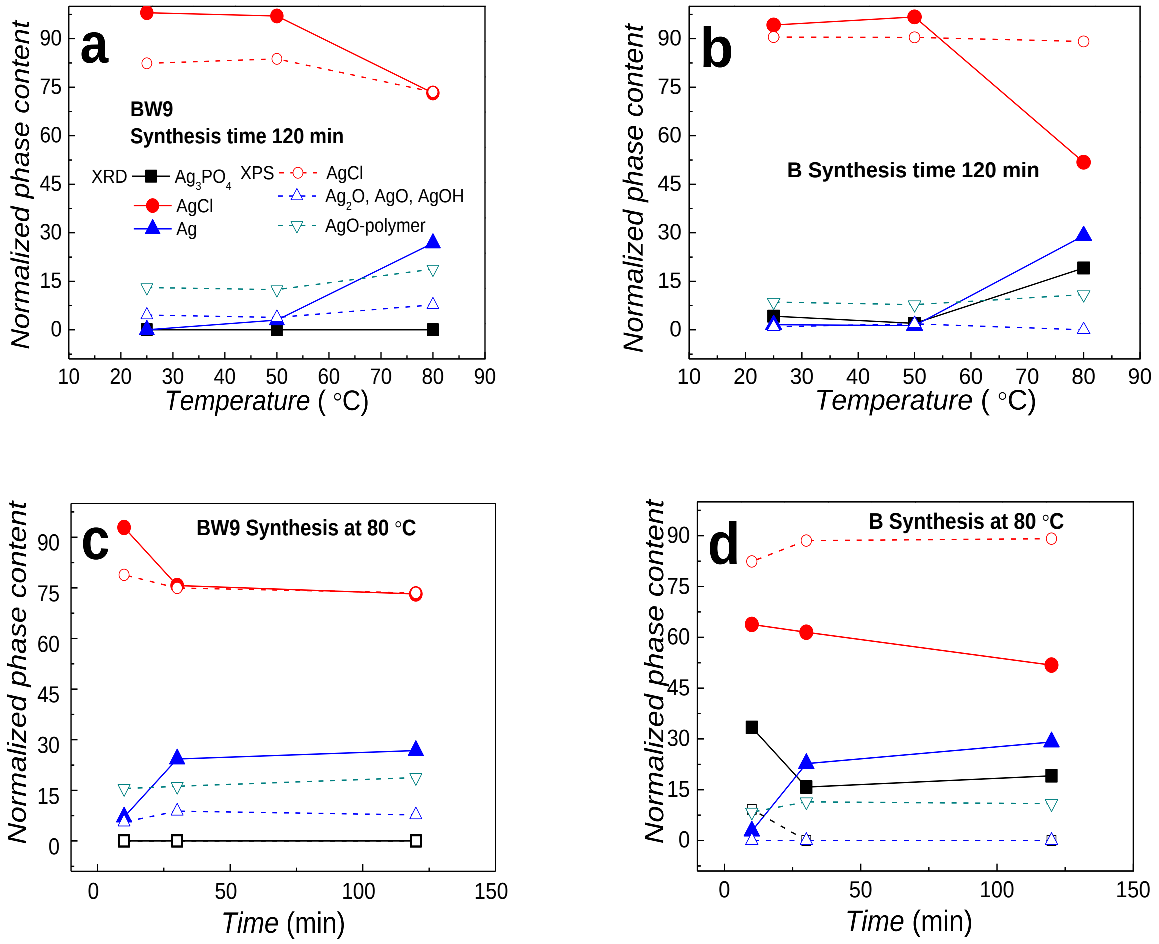

Figure S6. Weight and normalized phase content comparison resulting from XRD and XPS spectra, respectively, in nanocomposites synthesized at different temperatures and times using (a,c) brewery waste BW9 and (b,d) product B.

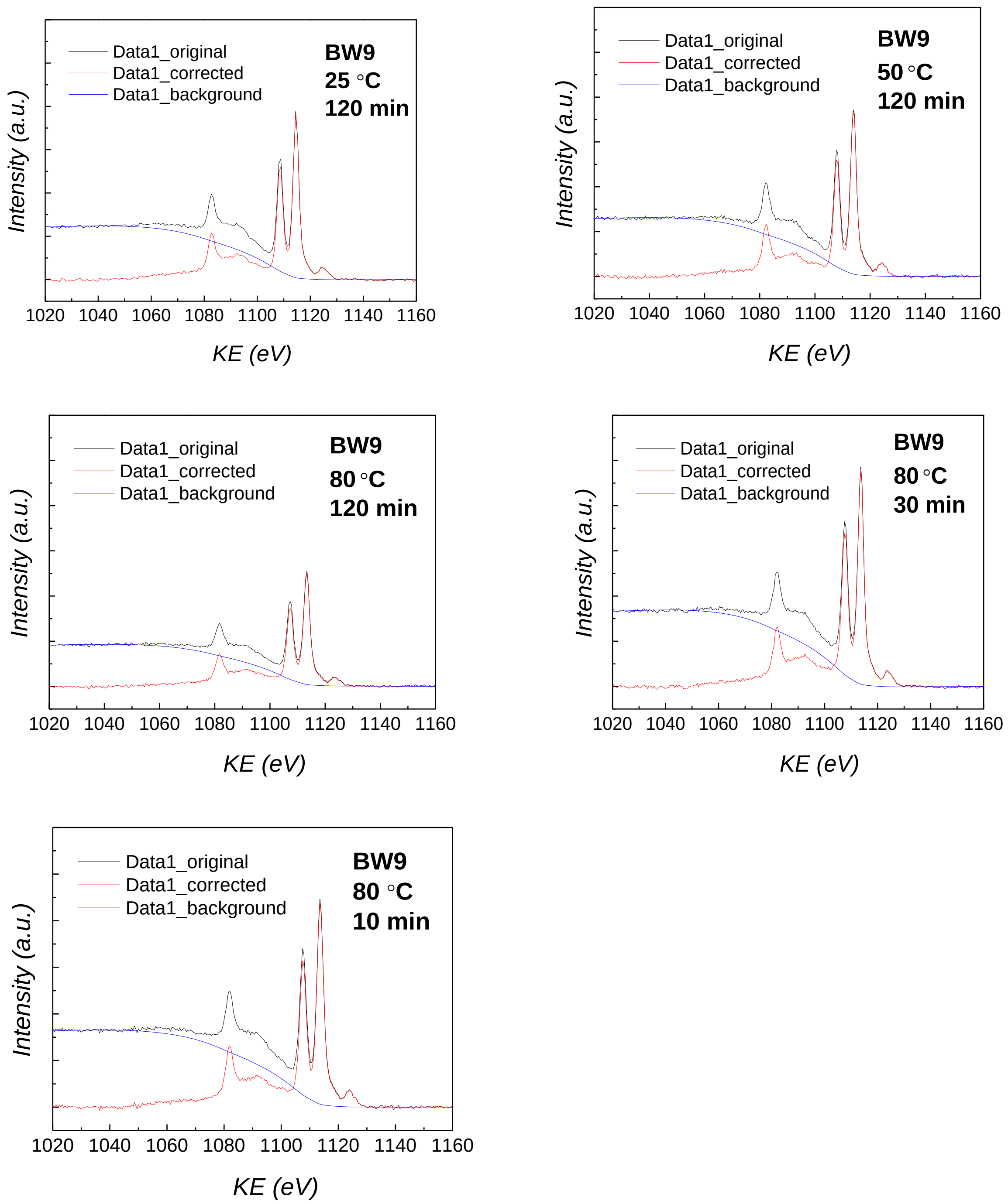

Figure S7. a. QUASES-Analyze software and Buried Layer (BL) model analysis of Ag $3d_{5/2/3-2}$ spectra for BW9 nanomaterials at different synthesis temperatures and times at 80 °C.

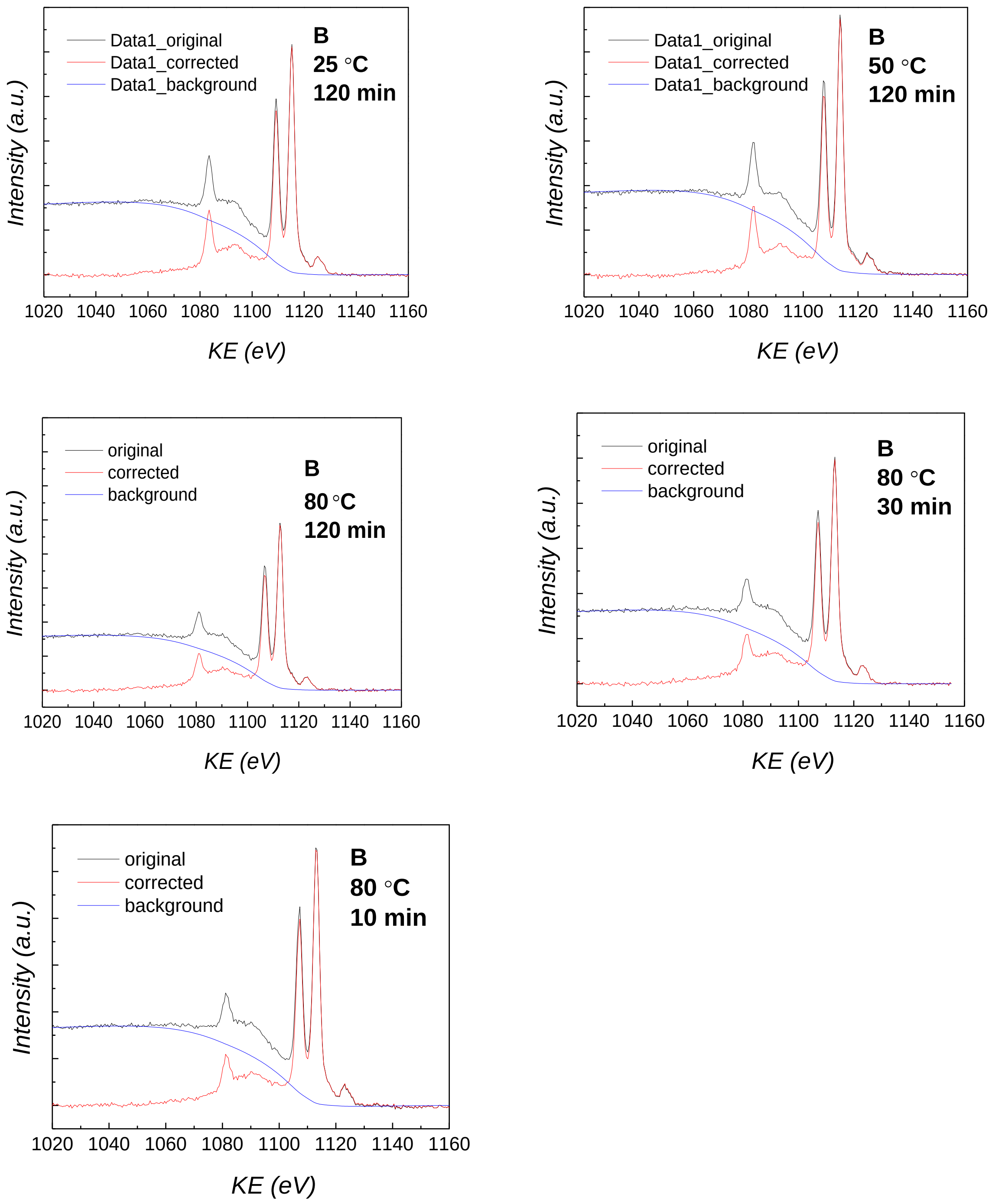

Figure S7. b. QUASES-Analyze software and Buried Layer (BL) model analysis of Ag $3d_{5/2/3-2}$ spectra for B nanomaterials at different synthesis temperatures and times at 80 °C.
